# Subunit Interaction Differences Between the Replication Factor C Complexes in *Arabidopsis* and Rice

**DOI:** 10.3389/fpls.2018.00779

**Published:** 2018-06-19

**Authors:** Yueyue Chen, Jie Qian, Li You, Xiufeng Zhang, Jinxia Jiao, Yang Liu, Jie Zhao

**Affiliations:** State Key Laboratory of Hybrid Rice, College of Life Sciences, Wuhan University, Wuhan, China

**Keywords:** *Arabidopsis*, DNA replication, protein interaction, rice, replication factor C

## Abstract

Replication factor C (RFC) is a multisubunit complex that opens the sliding clamp and loads it onto the DNA chain in an ATP-dependent manner and is thus critical for high-speed DNA synthesis. In yeast (*Saccharomyces cerevisiae*) and humans, biochemical studies and structural analysis revealed interaction patterns between the subunits and architectures of the clamp loaders. Mutations of *ScRFC1/2/3/4/5* lead to loss of cell viability and defective replication. However, the functions of RFC subunits in higher plants are unclear, except for *AtRFC1/3/4*, and the interaction and arrangement of the subunits have not been studied. Here, we identified *rfc2-1*/+, *rfc3-2*/+, and *rfc5-1*/+ mutants in *Arabidopsis*, and found that embryos and endosperm arrested at the 2/4-celled embryo proper stage and 6-8 nuclei stages, respectively. Subcellular localization analysis revealed that AtRFC1 and OsRFC1/4/5 proteins were localized in the nucleus, while AtRFC2/3/4/5 and OsRFC2/3 proteins were present both in the nucleus and cytoplasm. By using yeast two-hybrid (Y2H) and bimolecular fluorescence complementation (BiFC) techniques, we demonstrated the interactions of *Arabidopsis* and rice (*Oryza sativa*) RFC subunits, and proposed arrangements of the five subunits within the RFC complex, which were AtRFC5-AtRFC4-AtRFC3/2-AtRFC2/3-AtRFC1 and OsRFC5-OsRFC2-OsRFC3-OsRFC4-OsRFC1, respectively. In addition, AtRFC1 could interact with AtRFC2/3/4/5 in the presence of other subunits, while OsRFC1 directly interacted with the other four subunits. To further characterize the regions required for complex formation, truncated RFC proteins of the subunits were created. The results showed that C-termini of the RFC subunits are required for complex formation. Our studies indicate that the localization and interactions of RFCs in *Arabidopsis* and rice are distinctly discrepant.

## Introduction

In eukaryotes, the heteropentameric replication factor C (RFC) acts as a clamp loader that can bind and open the homotrimeric proliferating cell nuclear antigen (PCNA) clamp, and then load PCNA onto a template-primer junction in an ATP-dependent reaction (Fien and Stillman, 1992; O'Donnell and Kuriyan, 2006). Coupled with ATP hydrolysis, RFC is ejected from the PCNA clamp for the next round of DNA synthesis and the clamp recruits DNA polymerases for processive elongation of DNA chains (Yao and O'Donnell, 2012). In addition to its role in DNA replication, RFC has been reported to function in DNA polymerase switching (Maga et al., 2000; Mossi et al., 2000), DNA repair (Shivji et al., 1995; Waga and Stillman, 1998), and check-point control in cell cycle progression (Sugimoto et al., 1997; Shimada et al., 1999; Kim and Brill, 2001).

The RFC complex consists of five subunits: the large subunit is named RFC1 and four small subunits are named RFC2/3/4/5 (Yao and O'Donnell, 2012). The five subunits exist in all eukaryotes and are highly conserved in both structure and function (Chen et al., 1992; Bunz et al., 1993; Luckow et al., 1994; Cullmann et al., 1995; Gray and MacNeill, 2000; Furukawa et al., 2003). All five subunits have high homology to each other (Cullmann et al., 1995). Human RFCs were first identified as an essential replication factor for simian virus 40 (SV40) DNA replication *in vitro* (Tsurimoto and Stillman, 1989), and are composed of five subunits–p140, p40, p38, p37, and p36–whose molecular masses are 128.3, 39, 40.5, 39.6, and 38.5 kDa, respectively (Mossi and Hübscher, 1998). In yeast (*Saccharomyces cerevisiae*), a functional protein complex homologous to human RFC has been identified, which also consists of five subunits with molecular masses of 94.9, 39.9, 39.7, 38.2, and 36.2 kDa (Cullmann et al., 1995; Mossi and Hübscher, 1998). The yeast RFC subunits show high identity with human RFCs (Cullmann et al., 1995). RFC subunits possess a cluster of conserved motifs that are termed RFC boxes (Mossi and Hübscher, 1998). The four small subunits contain seven conserved RFC boxes II-VIII, which mainly exist in the N-terminal region. In addition to RFC boxes II-VIII, the N-terminal extension of the large subunit RFC1 contains an additional RFC box (box I), which shows high homology to the prokaryotic DNA ligases but lacks ligase activity (Kobayashi et al., 2010). RFC box III contains the most conserved motif: a phosphate-binding loop (P loop) that is essential for the structure and function of RFC (Cullmann et al., 1995; Podust et al., 1998; Neuwald et al., 1999; Schmidt et al., 2001). The C-terminal sequences of RFC subunits are flexible and essential for mediating subunit-subunit interaction as well as forming the integrated RFC complex (Shiomi and Nishitani, 2017).

In spite of the high sequence similarity and conserved structure, each subunit is indispensable for RFC complex formation and activity. In humans, deletion mutations of hRFC1/2/3/4/5 have shown that the C-terminal regions are indispensable for RFC complex formation (Uhlmann et al., 1997a,b). In yeast, all five subunits are essential for cell viability. Cold-sensitive *cdc44* (*rfc1*) mutants exhibited a delay in progressing through the S phase and arrested at the G2/M phase, and were sensitive to DNA damaging agent methyl methanesulfonate (MMS) and ultraviolet (UV) irradiation. Moreover, mutation of *POL30* (PCNA) suppressed cold-sensitive alleles of *cdc44* but could not substitute for its function. These results indicate that ScRFC1 plays important roles in both DNA replication and DNA repair (Howell et al., 1994; McAlear et al., 1994, 1996). Mutation of *ScRFC2* led to defects in DNA integrity and a disordered S-phase checkpoint, indicating that *ScRFC2* was required for chromosomal DNA replication and S-phase checkpoint control. On the other hand, *RFC5* suppressed the phenotype of *rfc2* mutation in yeast and the *rfc2-1 rfc5-1* and *rfc2-1 cdc44-1* double mutants were synthetically lethal, implying that ScRFC2 interacted with ScRFC1 and ScRFC5 in the RFC complex during DNA replication (Noskov et al., 1998). Mutation of *ScRFC5* gave rise to incomplete DNA replication and led to defects in entering into mitosis, indicating that *ScRFC5* was also involved in DNA replication and the S-phase checkpoint (Sugimoto et al., 1996; Naiki et al., 2000). In *Arabidopsis*, RFC1 was homologous to the large subunit p140 of human RFC and played an important role in meiotic recombination and crossover formation, and DNA double-strand break repair during meiosis (Liu et al., 2010, 2013; Wang et al., 2012). The *rfc3-1* mutant exhibited hypersensitivity to salicylic acid and enhanced resistance to virulent oomycete *Hyaloperonospora arabidopsidis* (*H. a*.) Noco2, suggesting that *AtRFC3* negatively regulates systemic acquired resistance and has crucial functions in cell proliferation and DNA replication (Xia et al., 2009, 2010). Mutation of *AtRFC4* had severe defects in DNA replication and led to seed abortion and seedling lethality, indicating that *AtRFC4* is crucial for DNA replication (Qian et al., 2018).

Studies on the interactions and arrangement of the five subunits contribute to further understanding of the structure of the RFC complex and the precise roles of individual subunits in complex formation. The crystal structure of the *E. coli* γ complex, the bacterial homolog of eukaryotic RFC, reveals that the γ complex is arranged in a circular manner and the C-terminal domains form a tight circular collar to mediate the subunit-subunit interactions of the complex (Jeruzalmi et al., 2001). In yeast, biochemical studies and structural analysis revealed that the model of RFC subunits was arranged in the form of ScRFC5-ScRFC2-ScRFC3-ScRFC4-ScRFC1 (Yao et al., 2003; Bowman et al., 2004). In humans, p36 (RFC3), p37 (RFC2), and p40 (RFC4) form a stable core complex, which can unload PCNA, but cannot load PCNA onto DNA. The p36/p37/p40 complex binds cooperatively to p140 (RFC1) and p38 (RFC5) subunits and forms the functional five-subunit RFC complex (Uhlmann et al., 1996; Cai et al., 1997; Ellison and Stillman, 1998). Electron microscopic studies confirm that hRFCs are also aligned in a circle, similar to the *E. coli* γ complex structure (Shiomi et al., 2000).

Despite the abundant literature about the RFC complex in yeast and humans, the functions of the five RFC subunits in higher plants are still unclear and the interaction and arrangement of the five subunits have not been studied. In this study, homology and structural analysis of the five subunits in *Arabidopsis* and rice (*Oryza sativa*) were carried out. The interactions of these subunits were investigated by employing the yeast two hybrid (Y2H) and bimolecular fluorescence complementation (BiFC) techniques. To characterize the regions required for complex formation, a series of truncated RFC proteins were produced to detect interactions. In addition, the phenotypes of *rfc2-1*/+, *rfc3-2*/+, and *rfc5-1*/+ mutants were characterized in *Arabidopsis*, which showed that both embryo and endosperm development were defective. Our studies increase knowledge for understanding subunit interaction relationships of the RFC complex, and provide new clues for further studies of the biological function of each RFC subunit.

## Materials and methods

### Plant materials and growth conditions

*Arabidopsis thaliana* Columbia-0 (Col-0) was used as the wild type in this study. The *rfc2-1* (CS800312 or SAIL_6_C02), *rfc3-2* (SAIL_401_E05), and *rfc5-1* (SALK_029291) were obtained from the ABRC (Arabidopsis Biological Resource Center). The *Arabidopsis* plants and wild type *Nicotiana benthamiana* plants were grown in a chamber at Wuhan University at 22 ± 2°C with a 16-h light and 8-h dark cycle.

### Phylogenetic analysis

The AtRFC1/2/3/4/5 and OsRFC1/2/3/4/5 protein sequences were used to search for the homologs from other species using BLASTP. Multiple sequence alignments of the box III and full-length amino acid sequences were performed using ClustalX (1.83) (Takashi et al., 2009). The phylogenetic tree with the Neighbor–Joining algorithm was constructed through the MEGA4 program (Tamura et al., 2007).

### Complementation analysis

For complementing the *rfc2-1/*+ and *rfc5-1/*+ mutants, the full length genomic fragments of *AtRFC2* and *AtRFC5* including the promoters and coding sequences were amplified from wild-type genome using KOD-Plus-Neo DNA polymerase (Toyobo, Japan) and cloned into *pCambia1300* vector, and then introduced into *rfc2-1/*+ and *rfc5-1/*+ heterozygote mutants by the floral dip method (Clough and Bent, 1998). Primers used in the experiments were listed in Table S4.

### Ovule clearing

Fresh ovules of *Arabidopsis* were dissected from siliques using forceps and mounted in Hoyer's solution [chloral hydrate: glycerol: water, 8:1:2 (w/v/v)] for 30 min or 6–8 h depending on the embryo developmental stage (Chen et al., 2015). Then, the cleared ovules were observed and photographed with differential interference contrast microscopy (Olympus TH4-200 equipped with a CCD of a SPOT digital microscope camera).

### Quantitative real-time PCR analysis

Total RNA from various tissues was extracted by RNAiso Plus (TaKaRa, Japan). RNA was used to transcribe into cDNA using ReverTra Ace qPCR RT Kit (TOYOBO). Quantitative Real-Time PCR was carried out as described (Li et al., 2017) and the relative expression levels were analyzed according to the reported method (Ma and Zhao, 2010). The expression level of the *GAPDH* gene was used as reference for the mRNA level. Three or more independent biological replicates and three technical replicates of each sample were performed for quantitative PCR analysis. Primers used in the experiments were listed in Table S4.

### Subcellular localizations

To construct GFP fusion vectors with OsRFC1/2/3/4/5 and Venus fusion vectors with AtRFC1/2/3/4/5, the coding sequence of the 10 proteins were fused in-frame to the N terminus of the enhanced GFP and Venus coding sequence under the control of the *CaMV 35S* promoter in the *pCAMBIA1300-EGFP* and *pCAMBIA1300-Venus* vector, respectively. The constructs were introduced into *A. tumefaciens* strain GV3101 and transformed into tobacco (*Nicotiana benthamiana*) leaves simultaneously by the agrobacterium-mediated transformation. After 2 days of incubation, fluorescence imaging of the tobacco epidermal cell was observed under an Olympus FluoView FV1000 confocal microscopy. Meanwhile, the *35S::Venus, 35S::AtRFC1/2/3/4/5-Venus* transgenic plants and *35S::GFP, 35S::OsRFC2/3-GFP* transgenic callus cells were obtained for subcellular localization analysis. The excitation and emission wavelength for GFP and Venus were 488 and 505–530 nm as well as 514 and 526–600 nm, respectively. Primers used were listed in Table S4.

### Yeast two-hybrid

The full-length open reading frames (ORFs) of *AtRFC1/2/3/4/5* and *OsRFC1/2/3/4/5* were cloned into the *pGADT7* and *pGBKT7* vector separately. Y2H assay was performed as described in our lab (Deng et al., 2014). The results were repeated at least three times. Primers used were listed in Table S4.

### Construction of tandem expression vectors

To construct the tandem expression vectors of *Arabidopsis* RFC complex, the CDS (coding sequence) of *AtRFC2* (*Sal*I+*Kpn*I), *AtRFC3* (*Sal*I+*Sac*I), *AtRFC4* (*Sal*I+*Kpn*I), and *AtRFC5* (*Sal*I+*Kpn*I) were amplified from the cDNA sample of 2-weeks seedlings using KOD-Plus-Neo DNA polymerase (Toyobo, Japan). The above CDS were then inserted into *mpCAMBIA1300* vector to build *35S::AtRFC2-NOST, 35S::AtRFC3-NOST, 35S::AtRFC4-NOST*, and *35S::AtRFC5-NOST* single vectors, respectively. Afterwards, primer pairs of 35S-FP-Hind III and NOST-RP-HindIII, 35S-FP-EcoRI and NOST-RP-EcoRI were used to obtain *35S::AtRFC3-NOST-35S::AtRFC2-NOST, 35S::AtRFC4-NOST-35S::AtRFC2-NOST, 35S::AtRFC4-NOST-35S::AtRFC3-NOST, 35S::AtRFC2-NOST-35S::AtRFC5-NOST, 35S::AtRFC3-NOST-35S::AtRFC5-NOST*, and *35S::AtRFC4-NOST-35S::AtRFC5-NOST* dual vectors. Similarly, the *35S:: AtRFC3-NOST-35S::AtRFC2-NOST-35S::AtRFC5-NOST, 35S:: AtRFC4-NOST-35S::AtRFC2-NOST-35S::AtRFC5-NOST, 35S:: AtRFC4-NOST-35S::AtRFC2-NOST-35S::AtRFC3-NOST*, and *35S:: AtRFC4-NOST-35S::AtRFC3-NOST-35S::AtRFC5-NOST* triple vectors were constructed. Primers used were listed in Table S4.

### BiFC assays

The full-length ORFs of *AtRFC1/2/3/4/5* and *OsRFC1/2/3/4/5* and their truncated cDNA were inserted into the *pCAMBIA-SPYNE* and *pCAMBIA-SPYCE* vectors, respectively. The constructs were transferred into *Agrobacterium tumefaciens* strain GV3101 by a freeze–thaw method. The experimental procedure was performed as described previously (Sparkes et al., 2006; Strzalka et al., 2015). Fluorescent images of YFP were taken with an Olympus FluoView FV1000 confocal microscope to determine whether the two proteins interact with each other. The excitation and emission wavelength for YFP was 515 and 505–530 nm, respectively. Empty vectors of BiFC constructs were used as a negative control. The results were repeated at least three times. Primers used in this test were listed in Table S4.

### Accession numbers

The accession numbers used in the article are *AtRFC1* (At5g22010), *AtRFC2* (At1g63160), *AtRFC3* (At1g77470), *AtRFC4* (At1g21690), *AtRFC5* (At5g27740), *OsRFC1* (Os11g0572100), *OsRFC2* (Os12g0176500), *OsRFC3* (Os02g0775200), *OsRFC4* (Os04g0569000), and *OsRFC5* (Os03g0792600).

## Results

### RFC subunits are conserved in eukaryotes

**Figure 1:**
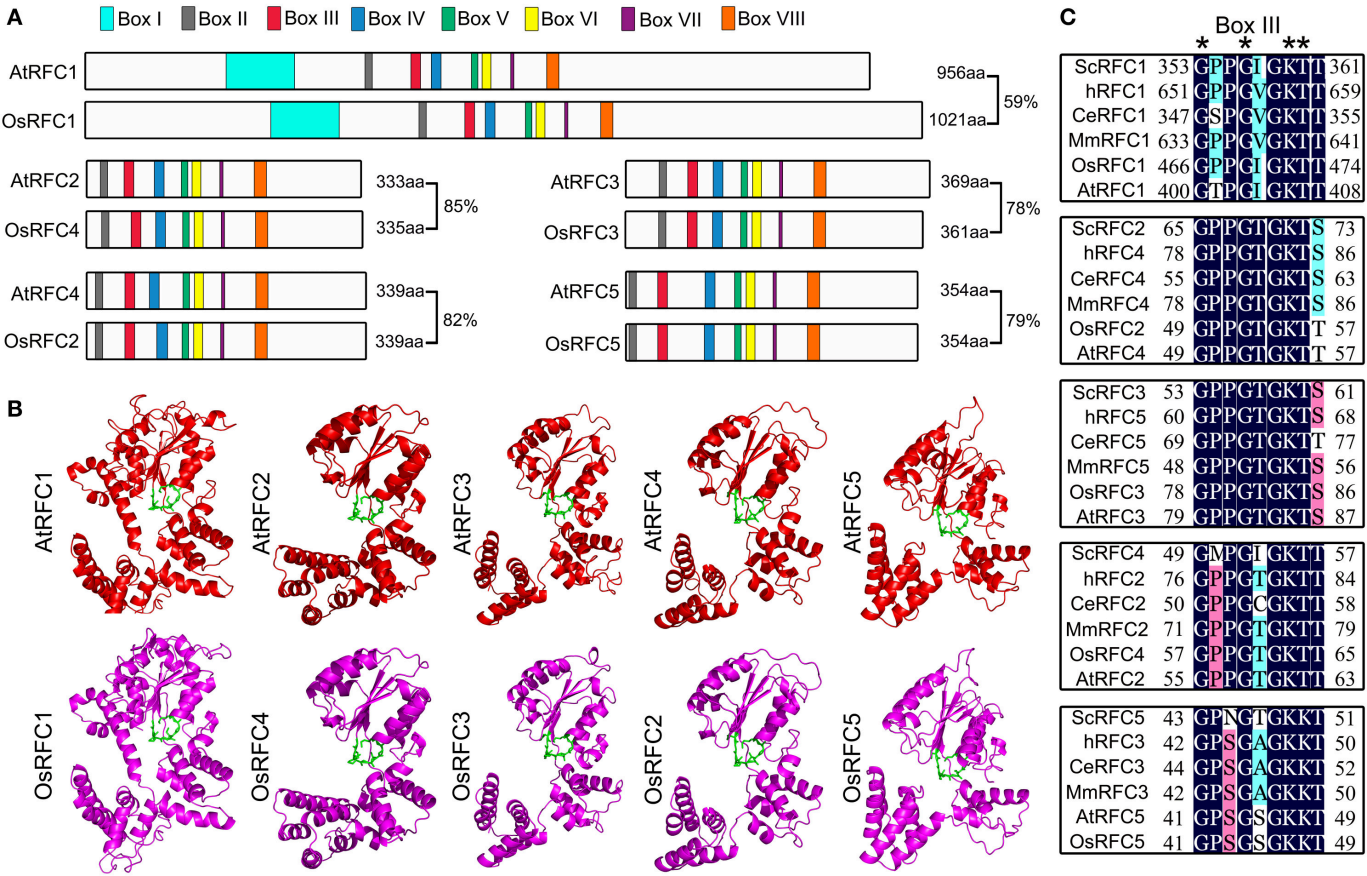
The conservation analysis of the five RFC subunits in *Arabidopsis* and rice. **(A)** Schematic diagram of the *Arabidopsis* and rice RFC subunits. AtRFC1 and OsRFC1 contain eight boxes I–VIII, while AtRFC2/3/4/5 and OsRFC2/3/4/5 contain seven boxes II-VIII. The length of the amino acid sequence is indicated at the end of each gene. **(B)** The three-dimensional structures of RFC subunits are modeled according to the crystal structures of their yeast homologs through SWISS-MODEL (https://www.swissmodel.expasy.org/). The potential ATP-binding domains (also known as box III) are colored in green. **(C)** Sequence alignment of the box III of the five subunits in different organisms. Asterisks indicate the conserved residues. At, *Arabidopsis thaliana*; Os, *Oryza sativa*; Sc, *Saccharomyces cerevisiae*; Ce, *Caenorhabditis elegans*; H, *Homo sapiens*; Mm, *Mus musculus*.

To determine the identity of RFC subunits between *Arabidopsis* and rice, we performed full alignment of the amino acid sequences. As shown in Figure 1A, the amino acid sequence of rice RFC subunits exhibited high identity with *Arabidopsis* ranging from 59 to 85%. Moreover, the RFC subunits shared highly conserved regions, defined as boxes I–VIII. The small RFC2/3/4/5 subunits contained RFC boxes II–VIII, while AtRFC1 and OsRFC1 had an additional box I in their N-terminal sequences. To gain insights toward the evolutionary relationships of RFC subunits in yeast, humans, *C. elegans*, mice, *Arabidopsis*, and rice, phylogenetic analyses were performed. The results indicated that the RFC complex was conserved in eukaryotes, and the *Arabidopsis* RFC proteins were closely related to the rice RFC subunits (Figure S1). To further study the conservation of RFC subunits of *Arabidopsis* and rice at the structure level, three-dimensional structures were constructed based on their homologs in yeast (Figure 1B). The models suggested that the rice RFC subunits exhibited extremely high similarity with *Arabidopsis* RFC subunits, providing further evidence that RFC structures were conserved in eukaryotes. As one of the most conserved motifs in RFC subunits, box III plays an essential role in ATP-binding in yeast and humans (Kelch, 2016). Sequence alignment of box III of the five subunits from *Arabidopsis*, rice and other eukaryotes revealed high sequence similarities and the conserved motif GxxxxGK (S/T) (Figure 1C).

### Knock-out of *AtRFC2/3/5* seriously inhibits the division of embryo cells and endosperm free nuclei

**Figure 2:**
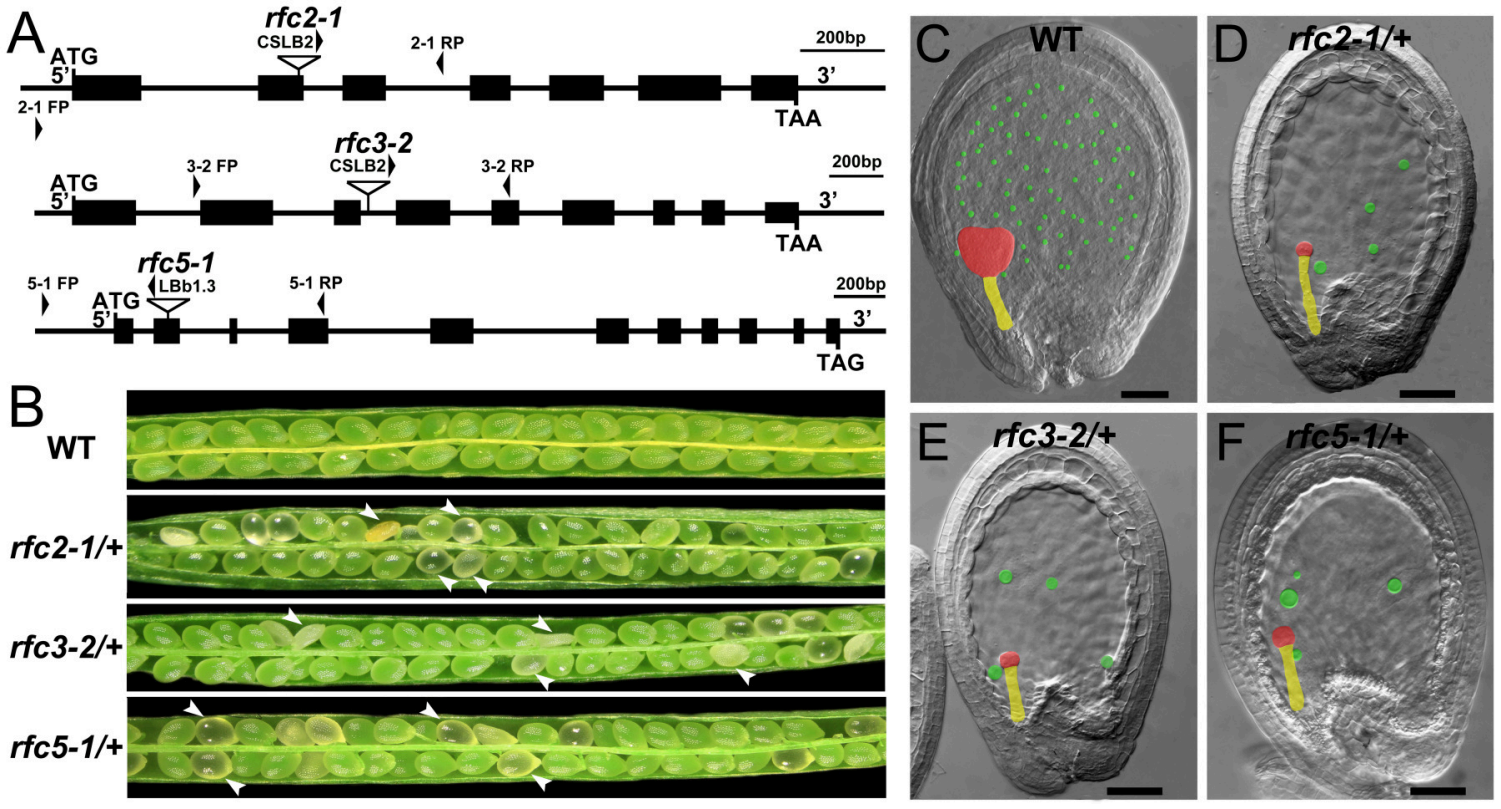
Characterization of *Arabidopsis rfc2-1/*+, *rfc3-2/*+, and *rfc5-1/*+ mutants. **(A)** Schematic diagrams of the *AtRFC2/3/5* genes and the positions of T-DNA insertions of their mutants. Exons are shown as black boxes, and 5′ regions, 3′ regions and introns as lines. Arrowheads indicate the positions of FP/RP primers used for genotyping. **(B)** Silique phenotypes of *Arabidopsis* wild type and *rfc2-1/*+, *rfc3-2/*+, and *rfc5-1/*+ mutants. The white arrowheads show the aborted white ovules. **(C–F)** Phenotypes of embryo and endosperm free nuclei in wild type, *rfc2-1/*+, *rfc3-2/*+, and *rfc5-1/*+ mutants. Red, yellow and green marks indicate the embryo proper, the suspensor and the endosperm free nuclei, respectively.

In *Arabidopsis*, the expression and function of *RFC1* and *RFC4* have been reported (Liu et al., 2013; Qian et al., 2018). To assess the functions of *AtRFC2/3/5*, we first detected their expressions in various *Arabidopsis* tissues using quantitative real-time PCR (qRT-PCR) assays. The results showed that *AtRFC2/3/5* were expressed in nearly all of the vegetative and reproductive tissues, and the expression levels were higher in the flowers and siliques (Figure S2). Meanwhile three T-DNA insertion mutants *rfc2-1, rfc3-2*, and *rfc5-1* were obtained from the Arabidopsis Biological Resource Center (ABRC). Primers were designed to identify the precise positions of the T-DNA insertions and sequencing showed that *rfc2-1* possessed a T-DNA in the second exon of *AtRFC2, rfc3-2* in the third intron of *AtRFC3* and *rfc5-1* in the second exon of *AtRFC5* (Figure 2A). We found that viable homozygotes in the progenies of the three mutant plants could not be obtained. Though no developmental defects were observed in heterozygous plants of the mutants during vegetative growth, their mature siliques contained white abnormal ovules at a frequency of about 25% (Figure 2B; Table 1). All of these results showed that the mutations in *AtRFC2/3/5* were lethal in homozygous progenies, similar to the mutant *rfc4* (Qian et al., 2018).

**Table 1:** The seed abortion ratios in the rfc2/3/5 mutants of *Arabidopsis*.

**Genotype**	**Normal (%)**	**Sterile (%)**	**n**
Wild type	99.36	0.64	1089
*rfc2–1/+*	74.62	25.38^**^	1174
*rfc3–2/+*	74.05	25.95^**^	921
*rfc5–1/+*	76.27	23.73^**^	1163

n, total number of seeds examined.

** *Significantly different from the abortion rates of wild type (P < 0.01)*.

To clarify whether the null mutation in *RFC2/3/5* affected gametophyte activity, segregation of the three self-fertilized mutant progenies were analyzed. The T-DNA insertion in *rfc2-1/*+ and *rfc3-2/*+ mutants harbors the Basta (Bas) resistance gene; segregation analysis of the mutant alleles was performed with resistance screening. Due to the lack of kanamycin resistance of the *rfc5-1/*+ mutant, its segregation was determined by PCR. All of the segregation ratios were about 2:1 (resistant:sensitive), instead of the expected 3:1 (Table S2). The results showed that the *rfc2-1/*+, *rfc3-2/*+, and *rfc5-1/*+ mutants contained a single copy T-DNA insertion in their respective genomes, and which led to aborted seeds in the mature siliques. We performed further reciprocal crosses with *rfc2-1/*+, *rfc3-2/*+, and *rfc5-1/*+ to wild-type plants, respectively, and the results showed that the transmission capacity of both females and males in the three mutants was normal (Table S3), indicating that knock out of *RFC2/3/5* genes did not affect the viability of gametophytes.

Detailed ovule phenotypes of *rfc2-1/*+, *rfc3-2/*+, and *rfc5-1/*+ were also investigated. At 4 days after pollination (DAP), all embryos in wild-type ovules had developed into late globular or heart stage (Figure 2C). However, in aborted ovules of the three mutants, all embryos arrested at the 2/4-celled embryo proper stage and the number of endosperm free nuclei decreased dramatically (Figures 2D–F; Table 2), indicating that embryo and endosperm development were severely delayed and repressed in the mutants. The result showed that endosperm proliferation was suppressed as early as the elongated zygote stage and finally reached 6-8 nuclei stages (Table 2). This finding suggested that *AtRFC2/3/5* played a crucial role in maintaining mitosis in early embryo cells and endosperm free nuclei.

**Table 2:** Distribution of endosperm free nuclei in the *rfc2-1/*+, *rfc3-2/*+, and *rfc5-1/*+ aborted ovules at sequential development stages.

**Name**	**Embryo stages**	**The frequency of endosperm free nuclei (%)**						**AVE**	**n**
		**1–4**	**5–8**	**9–16**	**17–32**	**33–48**	**49–72**		
Wild type	EZ	–	38.3	53.4	8.3	–	–	12.0	60
*rfc2–1/+*		100	–	–	–	–	–	3.3	60
*rfc3–2/+*		100	–	–	–	–	–	3.0	43
*rfc5–1/+*		100	–	–	–	–	–	2.6	62
Wild type	1–cell	–	–	15.0	85.0	–	–	24.4	60
*rfc2–1/+*		67.0	33.0	–	–	–	–	4.2	88
*rfc3–2/+*		52.5	47.5	–	–	–	–	4.4	40
*rfc5–1/+*		85.9	14.1	–	–	–	–	3.5	71
Wild type	2/4–cell	–	–	3.3	43.3	50.0	3.4	34.3	60
*rfc2–1/+*		32.1	64.1	3.8	–	–	–	5.3	53
*rfc3–2/+*		40.0	56.7	3.3	–	–	–	5.3	60
*rfc5–1/+*		69.1	30.9	–	–	–	–	3.9	55

*EZ, ovules at elongated zygote stage; 1-cell, ovules at one cell embryo proper stage; 2/4-cell, ovules at two or four cell embryo proper stage; AEN, average endosperm nucleus in single ovule; n, number of total seeds examined*.

To verify that the *rfc2-1/*+ and *rfc5-1/*+ phenotypes were caused by mutation in the *AtRFC2* and *AtRFC5* gene, respectively, we performed complementation of the two mutants by transforming the full-length genomic sequence of *AtRFC2* and *AtRFC5* into the *rfc2-1/*+ and *rfc5-1/*+ plants. Homozygous mutants were obtained from the progenies of the transgenic plants through screening, showing that their seed abortion phenotypes were rescued (Figures S3A,B). The results indicated that mutation of *AtRFC2/5* genes is responsible for the seed defective phenotype of *rfc2-1/*+ and *rfc5-1/*+ mutants.

### Subcellular localization of the *Arabidopsis* and rice RFC subunits

**Figure 3:**
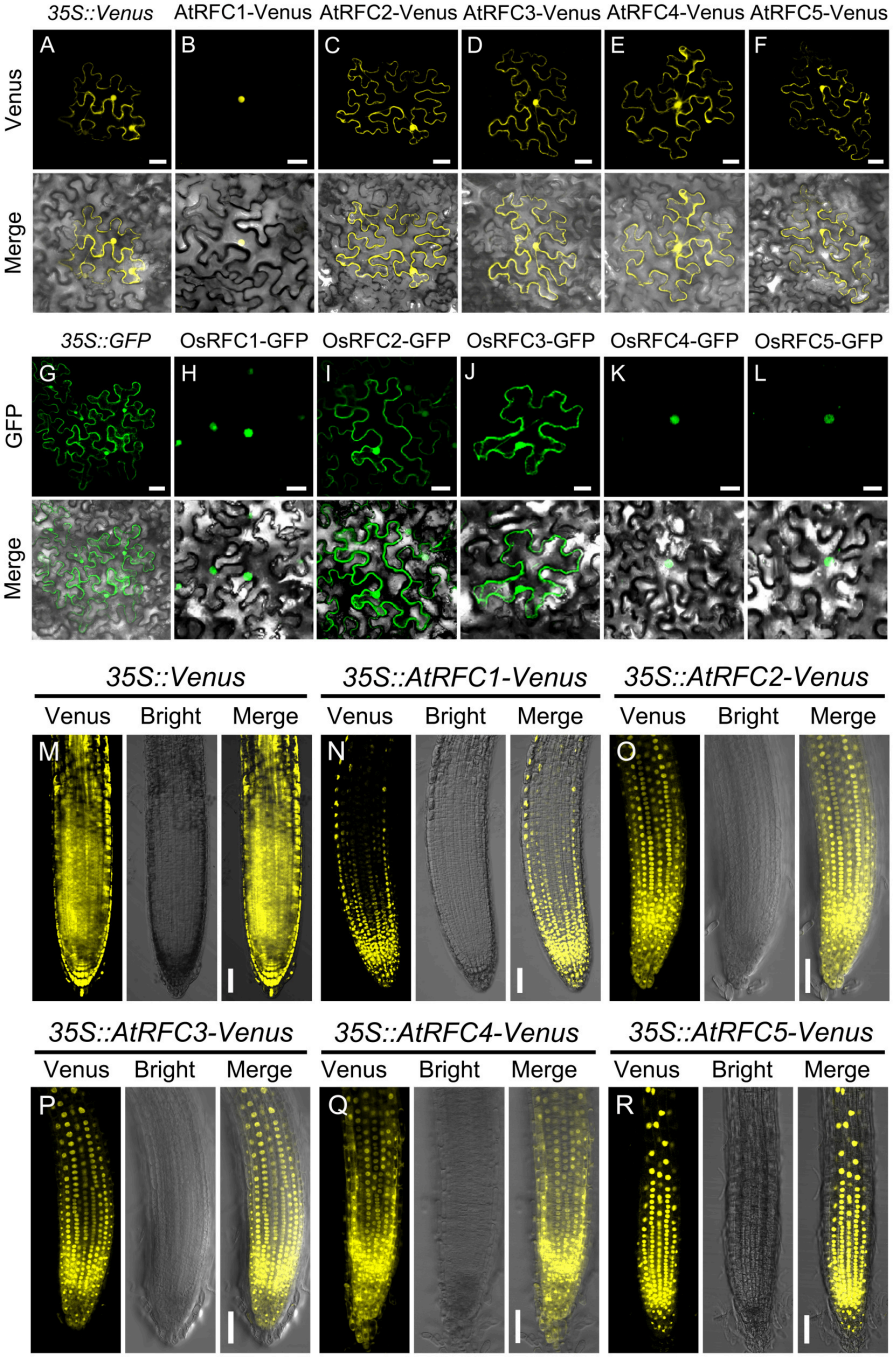
Subcellular localization of AtRFC1/2/3/4/5 and OsRFC1/2/3/4/5. **(A–F)** The subcellular localization of AtRFC1/2/3/4/5 in epidermic cells of the transiently-expressed *Nicotiana Benthamiana*. Bars = 30 μm. **(G–L)** The subcellular localization of OsRFC1/2/3/4/5 in epidermic cells of transiently-expressed *Nicotiana Benthamiana*. Bars = 30 μm. **(M–R)** The subcellular localization of AtRFC1/2/3/4/5 in root tip cells of the stably transformed *Arabidopsis* plants. Bars = 50 μm.

To characterize the subcellular localization of AtRFCs and OsRFCs, vectors expressing fusion proteins of AtRFC1/2/3/4/5 Venus and OsRFC1/2/3/4/5-GFP under the control of the *CaMV* 35S promoter were generated and expressed transiently in tobacco epidermal cells. The *35S::Venus* and *35S::GFP* vectors were used as controls, and their fluorescent signals were evenly distributed in the cytoplasm and the nucleus (Figures 3A,G). As previously reported (Liu et al., 2010), AtRFC1-Venus was preferentially localized in the nucleus (Figure 3B), whereas the fluorescent signals of AtRFC2/3/4/5-Venus fusion proteins accumulated in both the cytoplasm and the nucleus (Figures 3C–F). Consistent with AtRFC1-Venus, OsRFC1-GFP was also mainly localized in the nucleus (Figure 3H), while OsRFC2-GFP and OsRFC3-GFP were expressed ubiquitously (Figures 3I,J). Like OsRFC1, OsRFC4-GFP, and OsRFC5-GFP were only detected in the nucleus (Figures 3K,L). To further analyze the subcellular localization of AtRFCs and OsRFCs, we obtained the *35S::Venus, 35S::AtRFC1/2/3/4/5-Venus* transgenic plants and *35S::OsRFC2/3-GFP* transgenic callus cells, and observed their fluorescent signals. The results showed that the fluorescent signals of *35S::Venus* and *35S::GFP* were distributed in cytoplasm and nucleus (Figure 3M; Figure S4A). AtRFC1 was only localized in the nucleus of the root tip cells (Figure 3N). However, AtRFC2/3/4/5-Venus were mainly located in the nucleus, and a small amount in the cytoplasm of the root tip cells (Figures 3O–R). In rice callus cells, OsRFC2-GFP and OsRFC3-GFP were also mainly localized in the nucleus (Figures S4B,C). All of these results indicate that the RFC subunits were preferentially localized in the nucleus in *Arabidopsis* and rice.

### The subunit interactions of RFC in *Arabidopsis* and rice

**Figure 4:**
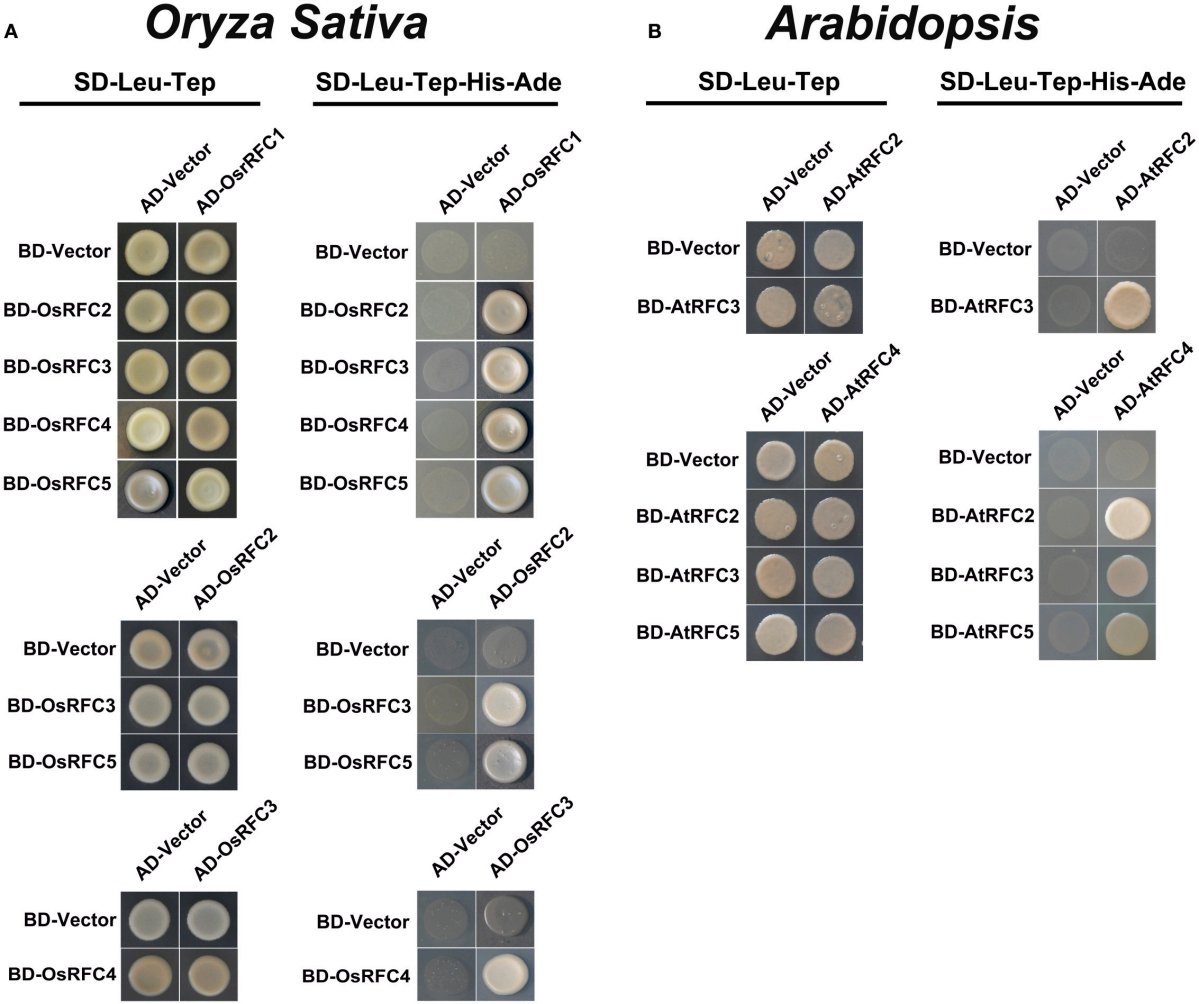
Yeast two-hybrid assay showing interactions among AtRFC2/3/4/5 subunits or OsRFC1/2/3/4/5 subunits. **(A)** Interactions between OsRFC1/2/3/4/5 subunits in rice. **(B)** Interactions between AtRFC2/3/4/5 subunits in *Arabidopsis*. Yeast two-hybrid assays were carried out and the co-transformed strains were spotted on SD-L-T/SD-L-T-H-A plates to test the direct interaction between the expressed proteins. Yeast strains co-transformed with the “empty” AD or BD plasmids were used as negative controls. AD, *pGADT7* vector; BK, *pGBKT7* vector; SD, synthetic dextrose; L, Leu; T, Trp; H, His; A, adenine.

Structural analyses of *E. coli* γ complex and yeast RFC complex revealed that the five subunits are arranged in a circular fashion (Jeruzalmi et al., 2001; Yao et al., 2003). However, it remains unclear how RFC subunits connect with each other to form an integrated RFC complex in higher plants. To investigate the subunit interactions among RFC subunits in *Arabidopsis* and rice, yeast two-hybrid assays were conducted. As shown in Figure 4A, OsRFC1 could interact with OsRFC2/3/4/5, OsRFC2 with OsRFC3/5, and OsRFC3 with OsRFC4, but no interaction was detected between OsRFC2 and OsRFC4. Meanwhile, it was found that AtRFC2, AtRFC3, and AtRFC4 could interact with each other, while AtRFC5 only interacted with AtRFC4 (Figure 4B). Unlike OsRFC1, AtRFC1 could not interact with AtRFC2/3/4/5 subunits.

**Figure 5:**
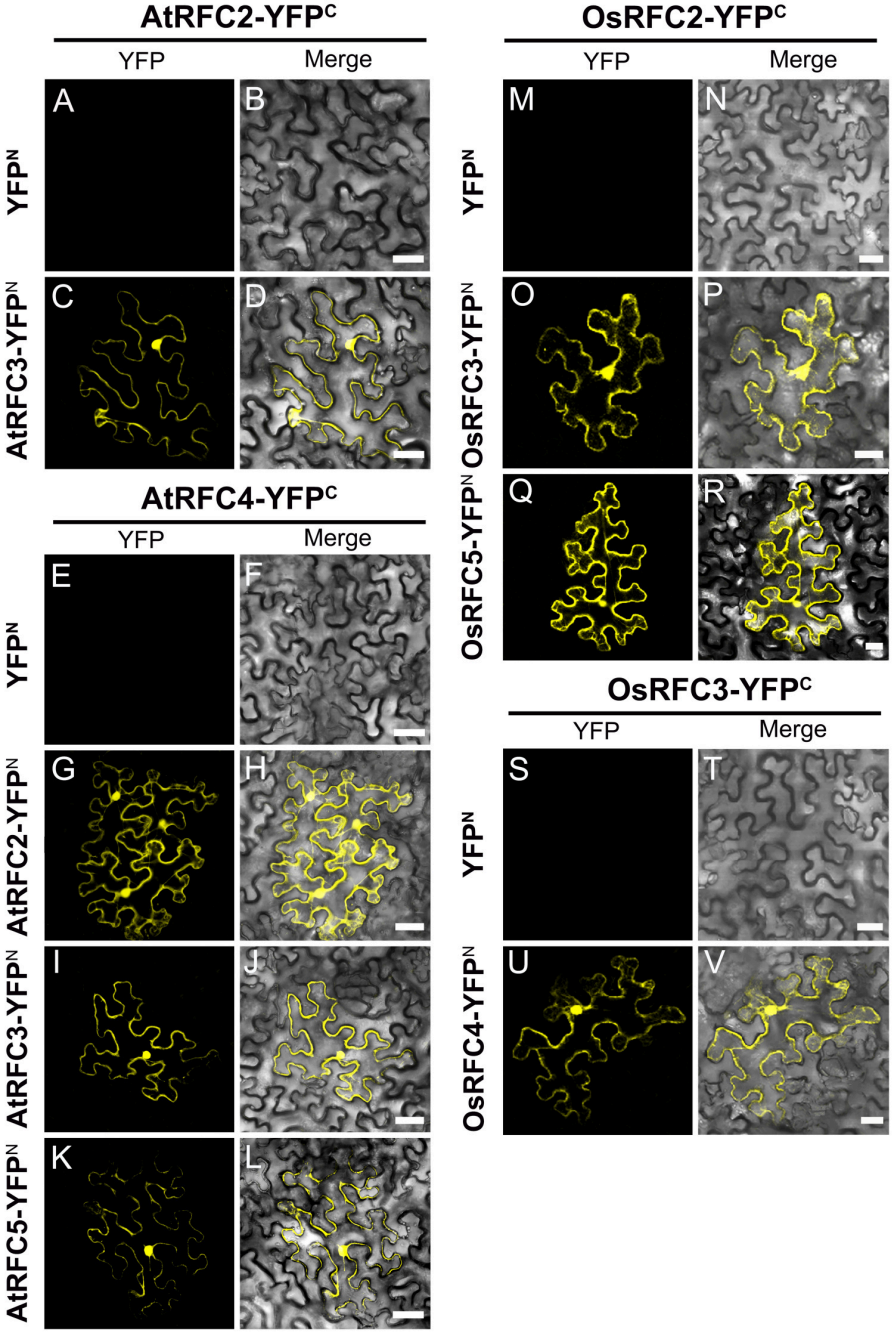
Analysis of interactions between AtRFC2/3/4/5 subunits or between OsRFC2/3/4/5 subunits using the BiFC assay in tobacco leaf epidermis cells. **(A–D)** AtRFC2 and AtRFC3 can interact with each other. **(E–L)** AtRFC4 can interact with AtRFC2/3/5, respectively. **(M–R)** OsRFC2 interacts with OsRFC3/5. **(S–V)** OsRFC3 interacts with OsRFC4. The tobacco epidermal cells were co-transfected with constructs encoding the indicated fusion proteins. YFP^C^, YFP C-terminal fragment (aa 156–239); YFP^N^, YFP N-terminal fragment (aa 1–155). Bars = 30 μm.

**Figure 6:**
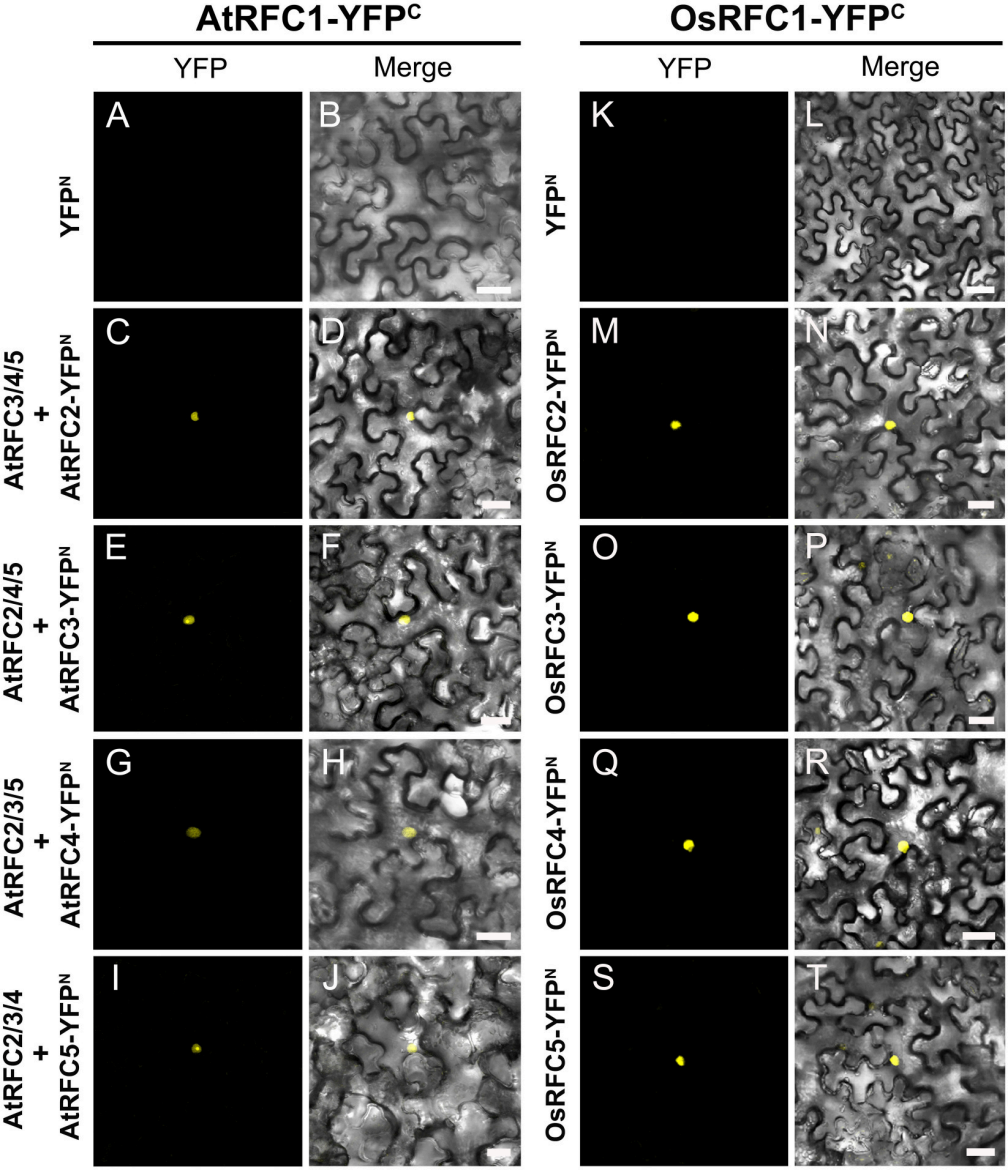
Analysis of the interactions between AtRFC1 and AtRFC2/3/4/5 as well as between OsRFC1 and OsRFC2/3/4/5 using the BiFC assay. **(A–J)** AtRFC1 can interact with AtRFC2/3/4/5 in the presence of all four AtRFC2/3/4/5 subunits in tobacco leaf cells. **(K–T)** OsRFC1 can directly interact with OsRFC2/3/4/5 in tobacco leaf cells. YFP^C^, YFP C-terminal fragment (aa 156–239); YFP^N^, YFP N-terminal fragment (aa 1–155). Bars = 30 μm.

Following the Y2H analysis, to further confirm the interactions of RFC subunits in *Arabidopsis* and rice, BiFC technique was employed. The N- and C-terminal ends of YFP protein were fused with AtRFCs and OsRFCs, respectively, and then the fusion proteins were co-expressed in tobacco leaves. As shown in Figure 5, no YFP signal was accumulated in epidermal cells co-transfected with YFP^N^ and AtRFC2-YFP^C^ or YFP^N^ and AtRFC4-YFP^C^ (Figures 5A,B,E,F). Obvious YFP signals were detected, however, in cells co-transfected with AtRFC3-YFP^N^ and AtRFC2-YFP^C^, AtRFC2/3/5- YFP^N^ and AtRFC4-YFP^C^ (Figures 5C,D,G–L), consistent with the results of Y2H assays, indicating that AtRFC2, AtRFC3, and AtRFC4 could interact with each other, while AtRFC5 interacted only with AtRFC4. On the other hand, YFP signals of OsRFC2-YFP^C^ and OsRFC3/5-YFP^N^, OsRFC3-YFP^C^ and OsRFC4-YFP^N^ were observed in the nucleus and cytoplasm of the transformed epidermal cells (Figures 5O–R,U,V), but there were no YFP signals between YFP^N^ and OsRFC2-YFP^C^ or YFP^N^ and OsRFC3-YFP^C^ (Figures 5M,N,S,T). Because AtRFC1 did not interact with any AtRFC2/3/4/5 subunits, it was speculated that the large subunit might be assembled into the complex with the assistance of other RFC subunits. Consistent with this presumption, YFP signals were observed in the nucleus of cells co-expressing AtRFC1-YFP^C^ and AtRFC2/3/4/5-YFP^N^ in the presence of all four AtRFC2/3/4/5 subunits (Figures 6C–J; Table S1), whereas YFP signals of OsRFC1-YFP^C^ and OsRFC2/3/4/5-YFP^N^ were only detected in the nucleus of the epidermal cells in the absence of RFC2/3/4/5 subunits (Figures 6M–T). No YFP signals were detected between YFP^N^ and AtRFC1-YFP^C^ or YFP^N^ and OsRFC1-YFP^C^ (Figures 6A,B,K,L). These results showed that the interaction patterns of AtRFCs were different from that of OsRFCs.

### Conserved substitution in the *Arabidopsis* and rice RFC subunits

Sequence alignment revealed that the RFC subunits in *Arabidopsis* and rice are highly conserved. To gain insights on the conserved nature of AtRFCs and OsRFCs, interaction assays between RFC subunits of *Arabidopsis* and rice were performed via BiFC in tobacco epidermal cells. According to the phylogenetic analysis (Figure S1), we exchanged AtRFC1 with OsRFC1, AtRFC2 with OsRFC4, AtRFC3 with OsRFC3, AtRFC4 with OsRFC2, and AtRFC5 with OsRFC5. The results showed that AtRFC1 could bind OsRFC2/3/4/5 when all four small RFC subunits existed simultaneously (Figures S5A–J), while OsRFC1 could directly interact with AtRFC2/3/4/5 in the absence of other AtRFCs subunits (Figures S5K–T), which was different from the interactions between AtRFC1 and AtRFC2/3/4/5. Likewise, OsRFC2 could interact with AtRFC2/3/5, respectively (Figures S6A–C). YFP signals of OsRFC3-YFP^N^ and AtRFC2/4-YFP^C^ were detected in the nucleus and cytoplasm of the tobacco epidermal cells (Figures S6D,E). YFP signals could also be observed in cells co-expressing OsRFC4-YFP^N^ and AtRFC3/4-YFP^C^ (Figures S6F,G). Moreover, OsRFC5 could directly interact with AtRFC4 (Figure S6H). Taken together, these results suggested that *Arabidopsis* and rice RFC proteins were highly conserved.

### The regions required for complex formation of the five *Arabidopsis* RFC subunits

In humans, deletion analysis of the large subunit p140 has shown that a region between amino acids 822 and 1,142 is required for the formation of the RFC complex (Uhlmann et al., 1997a). In the same way, sequences close to the C terminus of each of the small subunits were required for formation of the five-subunit complex (Uhlmann et al., 1997b). To identify the regions that were responsible for subunit interactions and complex formation of the five RFC subunits in *Arabidopsis* and rice, a series of truncated RFC proteins were fused with N- or C-terminus of the YFP and used in the BiFC assay.

**Figure 7:**
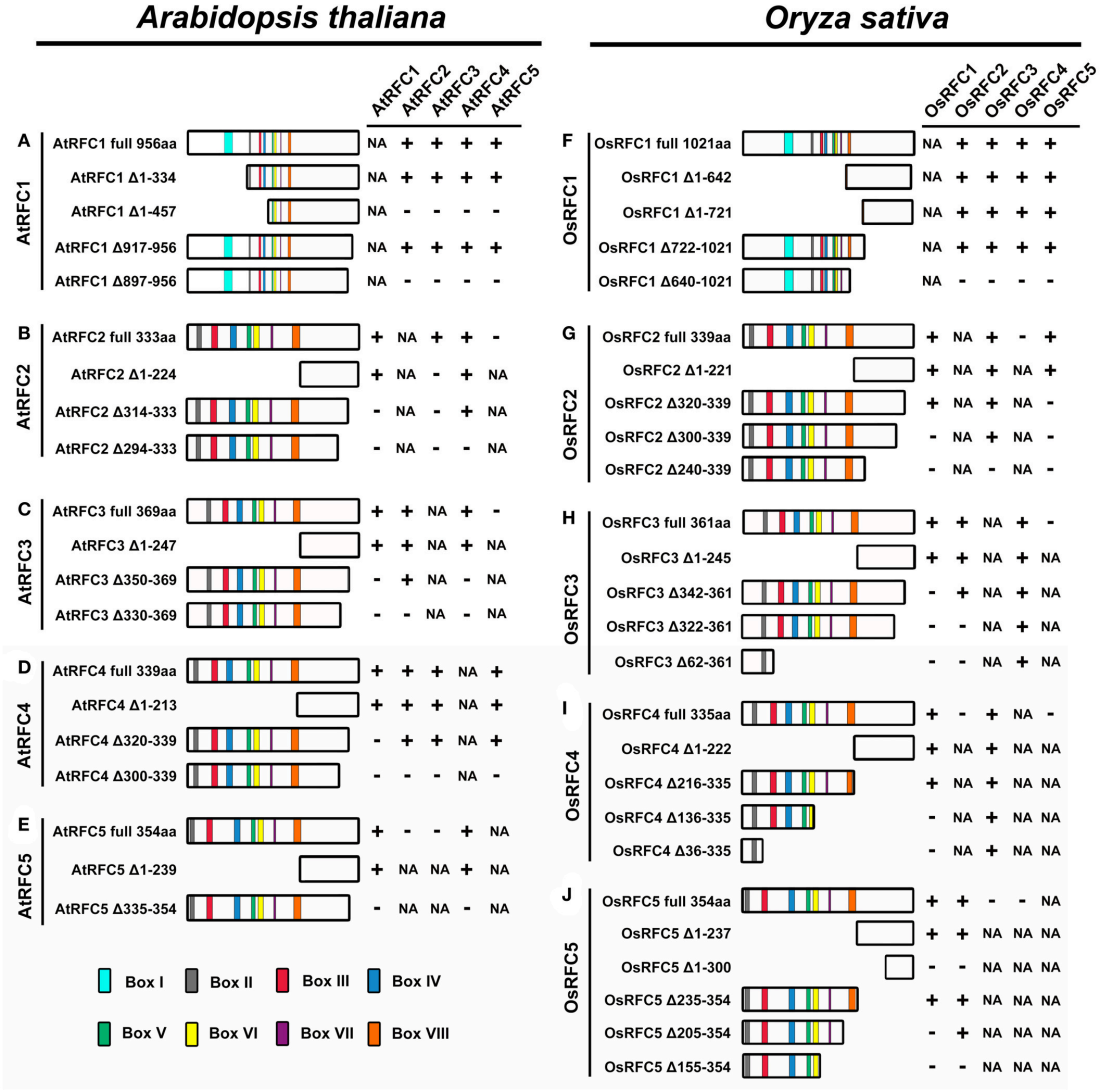
Summary of the truncated RFC subunits and their effects on RFC complex formation in *Arabidopsis* and rice. **(A–E)** Schematic diagrams of the regions required for RFC complex formation in *Arabidopsis*. **(F–J)** Schematic diagrams of the regions required for RFC complex formation in rice. The symbol “+” indicates that these proteins can interact; the symbol “–” indicates that these proteins cannot interact; the abbreviation NA indicates not applicable.

As shown in Figure 7A and Figure S7, the YFP signals of AtRFC1 Δ1-334-YFP^N^ and AtRFC2/3/4/5 YFP^C^ were observed in the nucleus of the transformed tobacco epidermal cells (Figures S7A–D), indicating that the region of amino acids 1–334 in AtRFC1 was not involved in the formation of the five-subunit RFC complex. However, no fluorescence signal was detected in cells co-expressing AtRFC1 Δ1-457-YFP^N^ and AtRFC2/3/4/5-YFP^C^ (Figures S7E–H), suggesting that the region of amino acids 335–457 of AtRFC1 was required for assembly of the RFC complex. On the other hand, we found that a deletion of 40 C-terminal amino acids (AtRFC1 Δ917-956-YFP^N^) did not affect interactions with AtRFC2/3/4/5 (Figures S7I–L), while an additional deletion of 20 C-terminal amino acids (AtRFC1 Δ897-956-YFP^N^) did not support the RFC complex formation (Figures S7M–P). These results indicated that the region within amino acids 335–457 and 898–917 of AtRFC1 mediated its interaction with AtRFC2/3/4/5 to form the five-subunit complex.

Deletion analysis of the AtRFC2 subunit is shown in Figure 7B and Figure S8. Deletion from the N terminus of AtRFC2 to amino acid 224 (AtRFC2 Δ1–224) did not affect the interactions between AtRFC2 and AtRFC1/4 (Figures S8A,C). AtRFC2 Δ314–333, with 20 C-terminal amino acids deleted, could interact with AtRFC4 (Figure S8F), but could not interact with AtRFC1 and AtRFC3 (Figures S8D,E). Deletion of an additional 20 amino acids at the C-terminus (AtRFC2 Δ294–333) led to a subunit variant that could not interact with AtRFC1/4 (Figures S8G,I). Moreover, the interactions between AtRFC2 and AtRFC3 disappeared no matter whether the N- or C-terminal sequence of AtRFC2 was truncated (Figures S8B,E,H). These results indicated that the C-terminal region between amino acids 314-333 of AtRFC2 mediated its interaction with AtRFC1, while the C-terminal region between amino acids 294–314 of AtRFC2 mediated its interaction with AtRFC4. In contrast to truncated AtRFC2, only the entire AtRFC2 protein could interact with AtRFC3.

Similar experiments were performed on deletion variants of the AtRFC3 subunit (Figure 7C; Figure S9). Full-length AtRFC3, as well as N-terminal deletion of AtRFC3 (AtRFC3 Δ1–247), supported interactions between AtRFC3 and AtRFC1/2/4 (Figures S9A–C). AtRFC3 Δ350–369, which lacked the 20 C-terminal amino acids no longer supported interactions between AtRFC3 and AtRFC1/4 (Figures S9D,F), but this did not affect its connection with AtRFC2 (Figure S9E). AtRFC3 ΔC330–369, which possessed an additional deletion of 20 C-terminal amino acids, did not interact with AtRFC1/2/4 (Figures S9G–I). These findings suggested that the C-terminal region of AtRFC3 between amino acids 350–369 was required for the interactions between AtRFC3 and AtRFC1/4, and the C-terminal region of AtRFC3 between amino acids 330–350 was required for interaction between AtRFC2 and AtRFC3.

Consistent with the results of AtRFC2/3, as a deletion variant of the AtRFC4 subunit, AtRFC4 Δ1–213 did not affect complex formation with AtRFC1/2/3/5, as the full-length AtRFC4 (amino acids 1–339) did (Figure 7D; Figures S10A–D). AtRFC4 Δ320–339, with 20 C-terminal amino acids deleted, supported interactions with AtRFC2/3/5, but did not support interaction with AtRFC1 (Figures S10E–H). When the full-length RFC4 subunit was replaced by RFC4 Δ300–339, a deletion protein lacking 40 C-terminal amino acids, its ability to interact with AtRFC1/2/3/5 was lost (Figures S10I–L). These results indicated that a C-terminal region of AtRFC4 between amino acids 320 and 339 was required for connection with AtRFC1, while the C-terminal region between 300 and 320 mediated the interactions between AtRFC4 and AtRFC2/3/5.

In the same way, we identified the regions responsible for complex formation of AtRFC5 (Figure 7E; Figure S11). The truncated protein lacking 239 N-terminal amino acids (AtRFC5 Δ1–239) did not affect its interactions with AtRFC1 and AtRFC4 (Figures S11A,B). However, when the 20 C-terminal amino acids of AtRFC5 (AtRFC5 Δ335–354) were deleted, the above interactions between AtRFC5 and AtRFC1/4 disappeared (Figures S11C,D). These results demonstrated that sequences from 335 to 354 of the C termini of AtRFC5 were required for the interactions with AtRFC1 and AtRFC4.

### Regions required for complex formation of the five rice RFC subunits

To investigate the regions that were required for complex formation in OsRFC1, deletion variants were used in the BiFC assay. The truncated proteins OsRFC1 Δ1–642 and OsRFC1 Δ1–721 that lacked 642 and 721 aa at the N-terminal end did not affect the interactions with the OsRFC2/3/4/5 (Figure 7F; Figures S12A–H). Similar results were observed when the region between 722 and 1,021 aa in the C-terminal of OsRFC1 was deleted (Figures S12I–L). However, OsRFC1 Δ640–1021, with another 82 C-terminus amino acids deleted, did not support RFC complex formation (Figures S12M–P). This indicated that a region between amino acids 722 and 1021 of OsRFC1 was sufficient for RFC complex formation and interactions between OsRFC1 and OsRFC2/3/4/5.

OsRFC2 Δ1–221, a variant containing a deletion of the boxes II-VIII was able to interact with OsRFC1/3/5 (Figure 7G; Figures S13A–C). Consistent with this, a deletion of 20 amino acids from the C terminus (OsRFC2 Δ320–339) did not affect the interaction with OsRFC1 or OsRFC3 (Figures S13D,E), but the connection with OsRFC5 disappeared (Figure S13F). Deletion of 40 C-terminal amino acids of OsRFC2 (OsRFC2 Δ300–339) resulted in a subunit variant incapable of supporting interaction with OsRFC1 or OsRFC5 (Figures S13G,I), while the deletion did not affect its ability to interact with OsRFC3 (Figure S13H). OsRFC2 Δ240–339, which lacked 100 C-terminal amino acids was unable to interact with OsRFC1/3/5 (Figures S13J–L). These results demonstrated that in the C terminus of OsRFC2, the region 320–339 mediated interaction with OsRFC5, the region 300–320 mediated interaction with OsRFC1, and the region 240–300 mediated interaction with OsRFC3.

Similar to OsRFC2, the truncated OsRFC3 Δ1–245 that lacked 245 aa N-terminal amino acids did not affect interactions with the other three subunits of OsRFC1/2/4 (Figure 7H; Figures S14A–C). OsRFC3 Δ342–361,with 20 C-terminal amino acids deleted, could interact with OsRFC2 and OsRFC4 but did not interact with OsRFC1 (Figures S14D–F). OsRFC3 Δ322–361, which possessed an additional deletion of 20 C-terminal amino acids, was able to interact with OsRFC4, but could not interact with OsRFC1 and OsRFC2 (Figures S14G–I). OsRFC3 Δ62–361, lacking 300 C-terminal amino acids, still interacted with OsRFC4, but not with OsRFC1 and OsRFC2 (Figures S14J–L). These results indicated that the regions between amino acids 342–361 and 322–342 were necessary for the interactions with OsRFC1 and OsRFC2, respectively. Different from the results of OsRFC1 and OsRFC2, the N-terminus and C-terminus of OsRFC3 were required for the interaction with OsRFC4.

Next, to identify the regions required for interactions with OsRFC1 or OsRFC3, deletion variants of OsRFC4 were prepared. OsRFC4 Δ1–222 that lacked 222 N-terminal amino acids, supported the interactions with OsRFC1 or OsRFC3 (Figure 7I; Figures S15A,B). Consistent with this, OsRFC4, with 120 amino acids deleted from the C terminus (OsRFC4 Δ216–335) was also able to interact with OsRFC1 or OsRFC3 (Figures S15C,D). However, OsRFC4 Δ136–335, with an additional 80 C-terminal amino acids deleted, did not support interaction with OsRFC1, but still interacted with OsRFC3 (Figures S15E,F). This indicated that a region between amino acids 136 and 216 within the OsRFC4 C-terminus was indispensable for interaction with OsRFC1, which was similar with the results of other small RFC subunits. OsRFC4 Δ36–335, with 300 C-terminal amino acids deleted, retained its ability to interact with OsRFC3 (Figure S15H), suggesting that the interaction between OsRFC4 and OsRFC3 might be very tight and thus was not affected by deleting N-terminal or C-terminal regions of OsRFC4.

Furthermore, N-terminal deletion of OsRFC5 that removed the 237 amino acids of its N-terminal end (OsRFC5Δ1–237) did not affect complex formation with OsRFC1 and OsRFC2 (Figure 7J; Figures S15I,J). However, when 300 N-terminal amino acids were deleted from of OsRFC5 (OsRFC5 Δ1–300), its ability to interact with OsRFC1 and OsRFC2 was lost (Figures S15K,L). A C-terminal deletion of the OsRFC5 that ended at amino acid 235 (OsRFC5 Δ235–354) did not affect its interactions with the OsRFC1 and OsRFC2 (Figures S15M,N). OsRFC5 Δ205–354, with an additional 30 C-terminal amino acids deleted, did not support interaction with OsRFC1 (Figure S15O), but still interacted with OsRFC2 (Figure S15P). OsRFC5 Δ155–354, with an additional 50 C-terminal amino acids deleted, did not support interaction with OsRFC1 and OsRFC2 (Figures S15Q,R). These results demonstrated that the region between amino acids 205 and 235 within the OsRFC5 C-terminus was indispensable for interaction with OsRFC1, while the amino acid sequences from 155 to 205 in its C terminus were required for interaction with OsRFC2.

### A model for subunit arrangement in RFCs

**Figure 8:**
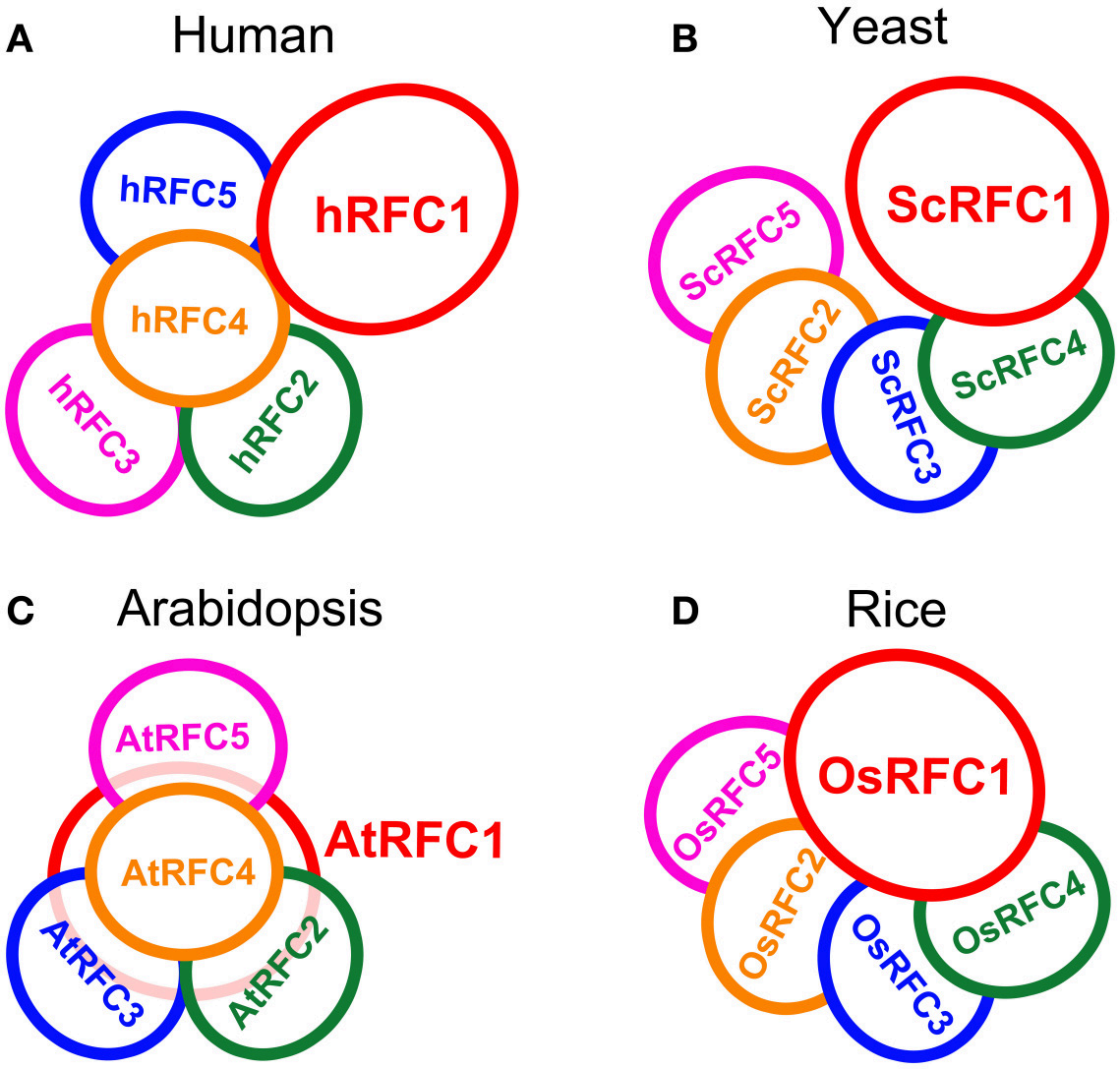
The models of RFC subunits arrangement in human **(A)**, yeast **(B)**, *Arabidopsis* **(C)**, and rice **(D)**.

Interaction models for the organization of the five subunits within the RFC complex were summarized on the basis of our studies in *Arabidopsis* and rice as well as the reported data in yeast and humans (Shiomi et al., 2000; Yao et al., 2003; Bowman et al., 2004). In humans, subunits p36, p37, and p40 form a three-subunit core complex where any two of the three subunits interact with each other; the p38 and p140 subunits cooperatively bind to the core complex in assembling the RFC complex (Figure 8A). The yeast RFC subunits assemble into a ring-shape structure with the arrangement ScRFC5-ScRFC2-ScRFC3-ScRFC4-ScRFC1 (Figure 8B). In *Arabidopsis*, as shown in Figure 8C, the subunits AtRFC2, AtRFC3, and AtRFC4 interacted with each other and formed a three-subunit sub-complex, AtRFC5 interacted with AtRFC4, and then AtRFC1 bound to the four-subunit complex assembling into a heteropentamer. The organization pattern of AtRFCs was AtRFC5-AtRFC4-AtRFC3/2-AtRFC2/3-AtRFC1, which was similar to those of humans, but the interactions between RFC1 and the other subunits were different. In rice, the interaction results provided a model where the subunits were arranged within the circular pentamer like OsRFC5-OsRFC2-OsRFC3-OsRFC4-OsRFC1 which was similar to yeast RFCs (Figure 8D), except that OsRFC1 could interact with the other four subunits. These results indicated that the organizations of the five RFC subunits were discrepant in different species, implying that the structure and function of different subunit interactions were diverse between human, yeast, and higher plants.

## Discussion

### The complex of five RFC subunits in *Arabidopsis* and rice may assemble in different pentameric forms

The clamp loaders composed of five distinct subunits are arranged in a circular shape in eukaryotes (Bowman et al., 2004). Biochemical analysis, electron microscopy and atomic force microscopy studies, and crystal structure of the RFC complex have provided detailed views of subunit interdigitates and the architecture of RFC in yeast and humans (Shiomi et al., 2000; Jeruzalmi et al., 2001; O'Donnell et al., 2001; Yao et al., 2003; Bowman et al., 2004). We summarized the interaction models of the RFC subunits within the complexes on the basis of the reported data in yeast and human as well as our studies in *Arabidopsis* and rice (Figure 8). In *Arabidopsis*, the arrangement of the subunits within the circular RFC complex was AtRFC5-AtRFC4-AtRFC3/2-AtRFC2/3-AtRFC1 (Figure 8C). The assembly of the five subunits may be initiated by formation of the RFC 2/3/4/5 tetramer, followed by recruitment of the large RFC1 to form a pentamer. The interaction pattern of the *Arabidopsis* RFC subunits was similar to that of human RFC (Figure 8A), but AtRFC1 can interact with the other four subunits in the presence of other subunits, while hRFC1 (p140) only interacts with hRFC4 (p40) and hRFC5 (p38) (Uhlmann et al., 1996; Cai et al., 1997; Ellison and Stillman, 1998). In yeast and rice, the models showed that the arrangements of the subunits were similar, namely RFC5-RFC2-RFC3-RFC4-RFC1 (Figures 8B,D). However, the difference was that OsRFC1 can directly interact with the other four subunits in rice, while ScRFC1 can interact only with ScRFC4 and ScRFC5 in yeast. Although the RFC complex forms a circular pentamer like $\gamma_{3}\delta \delta^{\prime }$ in eukaryotes, the organizations of the five subunits are discrepant in different species, indicating that the structure and arrangement of different subunit interactions are diverse from yeast and humans to higher plants.

### The C-terminal regions of the RFC subunits are required for complex formation

In humans, the five individual subunits were expressed though transcription/translation system and interacted to reconstitute a stable and bioactive complex with a three-subunit core complex. The large p140 (RFC1) interacted with the core complex in the presence of the p38 subunit (Cai et al., 1996, 1997; Podust and Fanning, 1997). Protein-protein interaction studies of yeast RFC showed that RFC2 interacted with RFC3/RFC5 and RFC3 interacted with RFC4 (Yao et al., 2003). In this study, we examined the interactions in the *Arabidopsis* and rice RFC subunits using Y2H and BiFC techniques. Our characterizations of the interactions between the *Arabidopsis* subunits were similar to the interaction pattern of the human subunits. AtRFC2, AtRFC3, and AtRFC4, like the p36p37p40 complex, interacted with each other and formed a three-subunit core complex. AtRFC1, like p140, binds to the core complex with the help of the other subunits. In rice, consistent with the interactions of the yeast subunits, the small subunits formed three sub-complexes –RFC2/3, RFC2/5 and RFC3/4–while OsRFC1 interacted with each of the other subunits.

To further investigate the regions within the five subunits responsible for complex formation, Y2H and BiFC assays were carried out using constructs of truncated RFC proteins. The ability of the truncated proteins of each subunit to form the RFC complex is summarized in Figure 7. The results showed that each subunit was required to form the RFC complex. The C-terminal regions of all *Arabidopsis* and rice RFC subunits were indispensable for subunit interactions. This was consistent with previous biochemical studies in humans and yeast as well as structural analysis of the *E. coli* and yeast clamp loaders (Uhlmann et al., 1996, 1997a,b; Jeruzalmi et al., 2001; Yao et al., 2003; Bowman et al., 2004). Different from the other subunits, in addition to the C-terminus, the N-terminal sequence of AtRFC2 was also required for the interaction with AtRFC3 (Figure 7B). Basically, the C-terminal regions mediated the strong interactions between individual subunits. In contrast, the truncated proteins lacking the conserved boxes close to their N-terminal regions did not affect the interactions with other subunits. This might explain why the C-terminal sequences of all five subunits are unique.

### Conservation and divergence of RFC subunits in *Arabidopsis* and rice

The clamp loading mechanism is functionally conserved across the domains of life (Trakselis and Benkovic, 2001; O'Donnell and Kuriyan, 2006). RFC subunits are evolutionarily conserved in both structure and function and exhibited significant sequence similarity to each other and to γ and δ′ of the *E. coli* γ complex (O'Donnell et al., 1993; Cullmann et al., 1995; Guenther et al., 1997). In particular, the RFC boxes II to VIII, which are mainly localized to the N-terminal half shared by the clamp loader, are highly conserved (Cullmann et al., 1995; Mossi and Hübscher, 1998). In this study, we analyzed and compared the structures and sequences of *Arabidopsis* and rice RFC complex (Figure 1). The molecular masses of AtRFC1 and OsRFC1 were 104.3 and 110.8 kDa, respectively. Similarly, the other four small subunits of *Arabidopsis* and rice possessed similar molecular mass, ranging from 36 to 42 kDa (Furukawa et al., 2003). In addition, the amino acid sequences of the five subunits in *Arabidopsis* showed a high degree of homology to the subunits of rice, up to 85%. The amino acid sequences of box III in the small subunits are similar as in their *Arabidopsis* counterpart. Although these subunits were homologous, it was not clear whether the rice subunits were the functional equivalent of the *Arabidopsis* orthologs. Thus, a substitution assay between *Arabidopsis* and rice was performed, and the results showed that every subunit played a similar role during the interaction among the subunits, implying that this complex was conserved in higher plants (Figures S5, S6).

Although most of the amino acids are highly conserved between *Arabidopsis* and rice RFC subunits, there are many variations in three-dimensional structure and sequence (Figure 1). For example, OsRFC1 has a longer N terminus compared with AtRFC1, and thus it is larger. The N-terminal extension of RFC1 is not required for cell viability and clamp loading activity but is related to DNA damage *in vivo* (Uhlmann et al., 1997a; Gomes et al., 2000). The P loop within box III is a general Walker-type ATPase motif with the consensus sequence GXXGXGKT. The residue Pro467 in box III of OsRFC1 corresponds to Thr401 in box III of AtRFC1. This subtle change may result in the differences in function. Consistent with the difference in structure and sequence, AtRFC1 can interact with AtRFC2/3/4/5 in the presence of the other subunits, while OsRFC1 can directly bind OsRFC2/3/4/5. For example, the mutation of four subunits in humans (not including p38) results in the Lys of the P-loop motif being replaced by Glu, which affects complex assembly and RFC function (Podust et al., 1998). The C-terminal regions of the RFC subunits in *Arabidopsis* required for subunit-subunit interactions were different from their counterparts in rice. Taken together, these differences may result in structural changes and may lead to divergence of functions between *Arabidopsis* and rice.

### RFC subunits are essential for cell viability in *Arabidopsis* and rice

Compared to the eukaryotic RFC clamp loader possessing four distinct small subunits, the gp44/62 complex in T4 bacteriophages possesses one large and four identical small subunits to load the clamp onto DNA (Moarefi et al., 2000). This indicates that there was some divergence between the clamp loaders in different organisms over evolutionary time, and the distinct small subunits of the RFC complex may have evolved diverse functions. In yeast, *ScRFC1* plays an essential role in DNA replication and DNA repair. Mutations in *ScRFC2, ScRFC3, ScRFC4, and ScRFC5* had defects in DNA replication and checkpoint controls, indicating that the subunits play crucial roles in DNA replication and the cell cycle checkpoint (Sugimoto et al., 1996, 1997; Noskov et al., 1998; Shimada et al., 1999; Gray and MacNeill, 2000; Kim and Brill, 2001). In *Drosophila, DmRfc4* mutants had reduced numbers of replicating cells and had defects in mitotic chromosomes and the cell cycle checkpoint, demonstrating that *DmRFC4* is essential for checkpoint control (Krause et al., 2001). In *Arabidopsis, AtRFC1* plays important roles in meiotic recombination and CO (crossover) formation, and DNA double-strand break repair during meiosis (Liu et al., 2010, 2013; Wang et al., 2012). *AtRFC3* plays a crucial role in systemic acquired resistance, cell proliferation and DNA replication (Xia et al., 2009, 2010). *AtRFC4* is crucial for DNA replication (Qian et al., 2018). In this study, we found that the *rfc2-1, rfc3-2*, and *rfc5-1* of the T-DNA insertion lines caused seed lethality (Figure 2A). Ovule clearing showed that all embryos arrested at the 2/4-celled embryo proper stage and the number of endosperm free nuclei decreased dramatically and finally endosperm reached 6–8 nuclei stages (Figures 2D–F; Table 2). The results suggested that *AtRFC2/3/5* were required for maintaining mitosis in early embryo cells and endosperm free nuclei. Our study and the previous reports suggested that RFCs have a variety of functions in addition to DNA replication in higher plants. Based on the conservation of the RFC complex in sequence, structure and function in loading the clamps onto DNA in different species, in particular *Arabidopsis*, we speculated that the rice RFC subunits also played important roles in DNA replication and DNA repair. In rice, the five RFC subunits are highly expressed in tissues where cell division is active and cell cycle inhibitors significantly reduced the expression of OsRFC5 and slightly affected the expression of OsRFC1/2/3/4 (Furukawa et al., 2003). These findings indicate that RFCs may be involved in cell proliferation and cell cycle progression. However, the specific functions of the *Arabidopsis* RFC2/3/5 subunits and rice RFC subunits are still being studied.

## Author contributions

YC performed most of the experiments, analyzed the research results and wrote the paper; JQ performed most of the experiments and analyzed the data; LY participated in Y2H and BiFC assays; XZ and JJ participated in BiFC assays; YL participated in temporal and spatial expressions of *AtRFC2/3/5*; JZ conceived the research plans, guided the whole study, and modified the paper.

### Conflict of interest statement

The authors declare that the research was conducted in the absence of any commercial or financial relationships that could be construed as a potential conflict of interest.
