# Supplementary material for: Subunit Interaction Differences Between the Replication Factor C Complexes in Arabidopsis and Rice
Source: Front Plant Sci. 2018 Jun 19;9:779. doi: 10.3389/fpls.2018.00779 (PMC6018503; doi:10.3389/fpls.2018.00779)
Supplement: Supplementary file 1 [file Table_1.doc]

**Subunit interaction differences between the replication factor C complexes in *Arabidopsis* and rice**

**Authors**: Yueyue Chen^†^, Jie Qian^†^, Li You, Xiufeng Zhang, Jinxia Jiao, Yang Liu, Jie Zhao^*^

**Address:** State Key Laboratory of Hybrid Rice, College of Life Sciences, Wuhan University, Wuhan 430072, China

***Corresponding author:** Jie Zhao

^†^ These authors contributed equally to this work.

**E-mail**: jzhao@whu.edu.cn

**Tel**: 86-27-68756010

**SUPPLEMENTARY MATERIAL**

**Table S1.** Interactions between AtRFC1 and AtRFC2/3/4/5 in the presence of the other subunits.

| experiment combinations | the results of interactions between AtRFC1 and AtRFC2/3/4/5 | | | | | | | |
| --- | --- | --- | --- | --- | --- | --- | --- | --- |
| AtRFC1-YC  AtRFC2-YN | AtRFC3 | + | + | + | - | + | - | - |
|  | AtRFC4 | + | + | - | + | - | + | - |
|  | AtRFC5 | + | - | + | + | - | - | + |
|  | Group | 3-4-5 | 3-4 | 3-5 | 4-5 | 3 | 4 | 5 |
|  | Results | + | - | - | - | - | - | - |
| AtRFC1-YC  AtRFC3-YN | AtRFC2 | + | + | + | - | + | - | - |
|  | AtRFC4 | + | + | - | + | - | + | - |
|  | AtRFC5 | + | - | + | + | - | - | + |
|  | Group | 2-4-5 | 2-4 | 2-5 | 4-5 | 2 | 4 | 5 |
|  | Results | + | - | - | - | - | - | - |
| AtRFC1-YC  AtRFC4-YN | AtRFC2 | + | + | + | - | + | - | - |
|  | AtRFC3 | + | + | - | + | - | + | - |
|  | AtRFC5 | + | - | + | + | - | - | + |
|  | Group | 2-3-5 | 2-3 | 2-5 | 3-5 | 2 | 3 | 5 |
|  | Results | + | - | - | - | - | - | - |
| AtRFC1-YC  AtRFC5-YN | AtRFC2 | + | + | + | - | + | - | - |
|  | AtRFC3 | + | + | - | + | - | + | - |
|  | AtRFC4 | + | - | + | + | - | - | + |
|  | Group | 2-3-4 | 2-3 | 2-4 | 3-4 | 2 | 3 | 4 |
|  | Results | + | - | - | - | - | - | - |
