# Supplementary material for: Subunit Interaction Differences Between the Replication Factor C Complexes in Arabidopsis and Rice
Source: Front Plant Sci. 2018 Jun 19;9:779. doi: 10.3389/fpls.2018.00779 (PMC6018503; doi:10.3389/fpls.2018.00779)
Supplement: Supplementary file 2 [file Table_2.doc]

**Subunit interaction** **differences between the replication factor C complexes in *Arabidopsis* and rice**

**Authors**: Yueyue Chen^†^, Jie Qian^†^, Li You, Xiufeng Zhang, Jinxia Jiao, Yang Liu, Jie Zhao^*^

**Address:** State Key Laboratory of Hybrid Rice, College of Life Sciences, Wuhan University, Wuhan 430072, China

***Corresponding author:** Jie Zhao

^†^ These authors contributed equally to this work.

**E-mail**: jzhao@whu.edu.cn

**Tel**: 86-27-68756010

**SUPPLEMENTARY MATERIAL**

**Table S2.** Segregation of the *AtRFC2/3/5* mutants in *Arabidopsis*

| Cross  (Female×Male)^a^ | W | WO | W:WO | Expected rate |
| --- | --- | --- | --- | --- |
| *rfc2-1/+* × *rfc2-1/+* | 1054 | 520 | 2.03:1^b^ | 3:1 |
| *rfc3-2/+* × *rfc3-2/+* | 947 | 468 | 2.02:1^b^ | 3:1 |
| *rfc5-1/+* × *rfc5-1/+* | 169 | 80 | 2.11:1^b^ | 3:1 |
| ^a^Seeds of each cross were grown on selective plates to determine the segregation for *rfc2-1/+* and *rfc3-2/+*; while seeds of each cross in *rfc5-1/+* were sown on nonselective plates and determined the segregation by PCR. ^b^Significantly different from the segregation ratio of 3:1 (P<0.01). WO, without T-DNA insert. W, with T-DNA insert. | | | | |
