# Supplementary material for: Subunit Interaction Differences Between the Replication Factor C Complexes in Arabidopsis and Rice
Source: Front Plant Sci. 2018 Jun 19;9:779. doi: 10.3389/fpls.2018.00779 (PMC6018503; doi:10.3389/fpls.2018.00779)
Supplement: Supplementary file 3 [file Table_3.doc]

**Subunit interaction** **differences between the replication factor C complexes in *Arabidopsis* and rice**

**Authors**: Yueyue Chen^†^, Jie Qian^†^, Li You, Xiufeng Zhang, Jinxia Jiao, Yang Liu, Jie Zhao^*^

**Address:** State Key Laboratory of Hybrid Rice, College of Life Sciences, Wuhan University, Wuhan 430072, China

***Corresponding author:** Jie Zhao

^†^ These authors contributed equally to this work.

**E-mail**: jzhao@whu.edu.cn

**Tel**: 86-27-68756010

**SUPPLEMENTARY MATERIAL**

**Table S3.** Transmission of the *AtRFC2/3/5* mutants in *Arabidopsis.*

| Cross  (Female×Male)^a^ | W | WO | W:WO | %TE_F_ | %TE_M_ | Expected  rate |
| --- | --- | --- | --- | --- | --- | --- |
| *rfc2-1/+* × WT | 528 | 543 | 0.97:1^b^ | 97.24 | NA | 1:1 |
| WT× *rfc2-1/+* | 519 | 496 | 1.05:1^b^ | NA | 104.64 | 1:1 |
| *rfc3-2/+* × WT | 480 | 498 | 0.96:1^b^ | 96.39 | NA | 1:1 |
| WT× *rfc3-2/+* | 538 | 544 | 0.99:1^b^ | NA | 98.89 | 1:1 |
| *rfc5-1/*+ × WT | 206 | 202 | 1.02:1^b^ | 101.98 | NA | 1:1 |
| WT × *rfc5-1/*+ | 215 | 208 | 1.03:1^b^ | NA | 103.36 | 1:1 |
| ^a^Seeds of each cross were grown on selective plates to determine the segregation for *rfc2-1/+* and *rfc3-2/+*; while seeds of each cross in *rfc5-1/+* were sown on nonselective plates and determined the segregation by PCR. ^b^Non-significantly different from the segregation ratio of 1:1 (P>0.05). WO, without T-DNA insert. W, with T-DNA insert. TE_F_, female transmission efficiency; TE_M_, male transmission efficiency; TE=Resistant/Sensitive × 100%. NA, not applicable. | | | | | | |
