# Supplementary material for: Subunit Interaction Differences Between the Replication Factor C Complexes in Arabidopsis and Rice
Source: Front Plant Sci. 2018 Jun 19;9:779. doi: 10.3389/fpls.2018.00779 (PMC6018503; doi:10.3389/fpls.2018.00779)
Supplement: Supplementary file 4 [file Table_4.doc]

**Subunit interaction** **differences between the replication factor C complexes in *Arabidopsis* and rice**

**Authors**: Yueyue Chen^†^, Jie Qian^†^, Li You, Xiufeng Zhang, Jinxia Jiao, Yang Liu, Jie Zhao^*^

**Address:** State Key Laboratory of Hybrid Rice, College of Life Sciences, Wuhan University, Wuhan 430072, China

***Corresponding author:** Jie Zhao

^†^ These authors contributed equally to this work.

**E-mail**: jzhao@whu.edu.cn

**Tel**: 86-27-68756010

**SUPPLEMENTARY MATERIAL**

Table S4. Primers (5' to 3') used in the experiments.

| 1.1 Primers for mutant verification | | |
| --- | --- | --- |
|  | FP | RP |
| *rfc2-1* | aattagggcattttgggtttg | aaagcgttgtgataatgtcgg |
| *rfc3-2* | aaatagtgaagagggag | gaaggtgttgttgtgattttga |
| *rfc5-1* | ttcctttcgtgtcaattggtc | aaataccattatccgatccgc |
| LBb1.3 | attttgccgatttcggaac |  |
| CSLB2 | gccttttcagaaatggataaatagc |  |
| 1.2 Primers for complementation | | |
|  | FP | RP |
| AtRFC2-Com | actGTCGACgatgagtcggacaaatcggg | cgcCTGCAGagtttacaccatttcacaat |
| AtRFC5-Com | cgcGGATCCtcaatggttggtactatcgc | cgcTCTAGAgcagactgataggccaaacc |
| PC1300 | cgggcctcttcgctattacg | aggcaccccaggctttacact |
| AtRFC2鉴定 | agttgagtttttttttgggtttg |  |
| AtRFC5鉴定 | aactatccgttcacagg |  |
| 1.3 Primers for RT-PCR | | |
|  | FP | RP |
| AtRFC2-RT | gattccttctcttccgact | gcttgtggttttacctgtt |
| AtRFC3-RT | tcactacattatcaagcacca | aataccaacaccttatatccc |
| AtRFC5-RT | tgccccatcttctgttgtat | gcgaaatcttgaatctgctg |
| 1.4 Primers for subcellular localization fusion constructs | | |
|  | FP | RP |
| OsRFC1-EGFP | gacTCTAGAatgtcttcggacatcaggaa | agaTCTAGAcctcttcctcttcccacctga |
| OsRFC2-EGFP | aacGGATCCatggcgccgctcgtgccg | ttaGGATCCgaagaccagcgtttgtggca |
| OsRFC3-EGFP | aacTCTAGAatggcgggagccaccgcc | gacGGATCCgtgggcagcagcaaccatag |
| OsRFC4-EGFP | agcTCTAGAatggacgcctccagctcctc | agaGGATCCtgatgcttttgctgtttctc |
| OsRFC5-EGFP | aacTCTAGAatgctgtgggtggacaagta | gacGGATCCgccaaatgtagagacaagaa |
| AtRFC1-EGFP | agaTCTAGAatgtcggatattaggaagtg | agaGGTACCtctctttctcttggcaccag |
| AtRFC2-EGFP | agaGGTACCatggcgtcttcttcatcaac | cgcGGATCCaggtgctttcgctgtttcac |
| AtRFC3-EGFP | agaTCTAGAatgactgagctaacgtcggc | cgcGGATCCctttgctgcaccaacaatga |
| AtRFC4-EGFP | cgcGGTACCatggcgccagttcttcagag | cgcGGATCCgaagtcttgagccatttctg |
| AtRFC5-EGFP | agaTCTAGAatgttgtgggtcgacaagta | cgcGGATCCcccaaacgttgaaatgagga |
| 1.5 Primers for yeast two-hybrid | | |
|  | FP | RP |
| OsRFC1-AD | cgGAATTCatgtcttcggacatcaggaaat | cGAGCTCGcctcttcctcttcccacctg |
| OsRFC1-BK | cgGAATTCatgtcttcggacatcaggaaat | gcGTCGACGcctcttcctcttcccacctg |
| OsRFC2-AD/BK | cgGAATTCatggcgccgctcgtgccg | cgGGATCCcgaagaccagcgtttgtggcatgtc |
| OsRFC3-AD/BK | cgGAATTCatggcgggagccaccgcc | cgGGATCCcgtgggcagcagcaaccatagcc |
| OsRFC4-AD/BK | cgGAATTCatggacgcctccagctcct | cgGGATCCctgatgcttttgctgtttctctca |
| OsRFC5-AD/BK | cgGAATTCatgctgtgggtggacaagta | cgGGATCCcgccaaatgtagagacaagaaatt |
| AtRFC1-AD | cgcGAGCTCgaatgtcggatattaggaag | cgcGAGCTCtctctttctcttggcaccag |
| AtRFC1-BK | agaCCCGGGaatgtcggatattaggaagt | agaCCCGGGtctctttctcttggcaccag |
| AtRFC2-AD/BK | cgcGAATTCatggcgtcttcttcatcaac | agaGGATCCcaggtgctttcgctgtttca |
| AtRFC3-AD/BK | cgcGAATTCatgttgtgggtcgacaagta | agaGGATCCccccaaacgttgaaatgagg |
| AtRFC4-AD/BK | cgcGAATTCatggcgccagttcttcagag | agaGGATCCcgaagtcttgagccatttctg |
| AtRFC5-AD/BK | cgcGAATTCatgactgagctaacgtcggc | agaGGATCCcctttgctgcaccaacaatg |
| 1.6 Primers for BiFC | | |
|  | FP | RP |
| OsRFC1-YC/YN | gcTCTAGAatgtcttcggacatcaggaaat | gcGTCGACcctcttcctcttcccacctg |
| OsRFC2-YC/YN | cgGGATCCatggcgccgctcgtgccg | ggGGTACCgaagaccagcgtttgtggcatgtc |
| OsRFC3-YC/YN | gcTCTAGAatggcgggagccaccgcc | cgGGATCCgtgggcagcagcaaccatagcc |
| OsRFC4-YC/YN | gcTCTAGAatggacgcctccagctcct | cgGGATCCtgatgcttttgctgtttctctca |
| OsRFC5-YC/YN | gcTCTAGAatgctgtgggtggacaagta | cgGGATCCgccaaatgtagagacaagaaatt |
| AtRFC1-YC/YN | agaGTCGACatgtcggatattaggaagtg | cgcGGTACCtctctttctcttggcaccag |
| AtRFC2-YC/YN | agaGTCGACatggcgtcttcttcatcaac | cgcGGTACCaggtgctttcgctgtttcac |
| AtRFC3-YC/YN | agaGTCGACatgactgagctaacgtcggc | cgcGGTACCctttgctgcaccaacaatga |
| AtRFC4-YC/YN | agaGTCGACatggcgccagttcttcagag | cgcGGTACCcactatgatagtctcataag |
| AtRFC5-YC/YN | cgcTCTAGAatgttgtgggtcgacaagta | cgcGGATCCcccaaacgttgaaatgagga |
| 1.7 Primers for construction of tandem expression vectors | | |
|  | FP | RP |
| AtRFC2-CDS | agaGGTACCatggcgtcttcttcatcaac | cgcGTCGACctaaggtgctttcgctgttt |
| AtRFC3-CDS | agaGTCGACatgactgagctaacgtcggc | cgcGAGCTCctactttgctgcaccaaca |
| AtRFC4-CDS | agaGGTACCatggcgccagttcttcagag | cgcGTCGACctacactatgatagtctcat |
| AtRFC5-CDS | agaGGTACCatgttgtgggtcgacaagta | cgcGTCGACctacccaaacgttgaaatg |
| 35S-FP-HindIII | agaAAGCTTtaaatgttcctcgctgacgt |  |
| 35S-FP-EcoRI | agaGAATTCtaaatgttcctcgctgacgt |  |
| NOST-RP-HindIII |  | cgcAAGCTTgatctagtaacatagatgac |
| NOST-RP-EcoRI |  | cgcGAATTCgatctagtaacatagatgac |
| 1.8 Primers for deletion analysis of RFC for BiFC | | |
|  | FP | RP |
| OsRFC1Δ1-642 | gcTCTAGAatgctctcccagtccgtggtca | gcGTCGACcctcttcctcttcccacctg |
| OsRFC1Δ1-721 | gcTCTAGAatggttaaaagaatgaattttct | gcGTCGACcctcttcctcttcccacctg |
| OsRFC1Δ722-1021 | gcTCTAGAatgtcttcggacatcaggaaat | gcGTCGACtccactgtcatccttcccaa |
| OsRFC1Δ640-1021 | gcTCTAGAatgtcttcggacatcaggaaat | gcGTCGACgactgggagaggctcataa |
| OsRFC2Δ1-221 | cgGGATCCatgttatttggatcttctattt | ggGGTACCgaagaccagcgtttgtggcatgtc |
| OsRFC2Δ320-339 | cgGGATCCatggcgccgctcgtgccg | ggGGTACCaagctgtaaatactcatccg |
| OsRFC2Δ300-339 | cgGGATCCatggcgccgctcgtgccg | ggGGTACCacatattcttgccttctgct |
| OsRFC2Δ240-339 | cgGGATCCatggcgccgctcgtgccg | ggGGTACCgatagcccctgaaacacta |
| OsRFC3Δ1-245 | gcTCTAGAatggcatctaagcaaataacag | cgGGATCCgtgggcagcagcaaccatagc |
| OsRFC3Δ342-361 | gcTCTAGAatggcgggagccaccgcc | cgGGATCCctgtaacttgtcattgcaag |
| OsRFC3Δ322-361 | gcTCTAGAatggcgggagccaccgcc | cgGGATCCcagctttatgcgtacatcag |
| OsRFC3Δ62-361 | gcTCTAGAatggcgggagccaccgcc | cgGGATCCggtgtcgacgatgtcgcggtg |
| OsRFC4Δ1-222 | gcTCTAGAatggggttccgttttgttaatc | cgGGATCCtgatgcttttgctgtttctctca |
| OsRFC4Δ216-335 | gcTCTAGAatggacgcctccagctcct | cgGGATCCgttcaaagcttgcctcatgt |
| OsRFC4Δ136-335 | gcTCTAGAatggacgcctccagctcct | cgGGATCCcagtgcttgctgtgctcccgat |
| OsRFC4Δ36-335 | gcTCTAGAatggacgcctccagctcct | cgGGATCCggagttgccgccgacgtcgg |
| OsRFC5Δ1-237 | gcTCTAGAatgcagcaatacccatttacat | cgGGATCCgccaaatgtagagacaagaaatt |
| OsRFC5Δ1-300 | gcTCTAGAatgttactgaagaaattagactc | cgGGATCCgccaaatgtagagacaagaaatt |
| OsRFC5Δ235-354 | gcTCTAGAatgctgtgggtggacaagta | cgGGATCCggtctcaaaaaacagtatcg |
| OsRFC5Δ205-354 | gcTCTAGAatgctgtgggtggacaagta | cgGGATCCtttcttcccaatgaactcca |
| OsRFC5Δ155-354 | gcTCTAGAatgctgtgggtggacaagta | cgGGATCCcattgtcctacgaagggaatgc |
| AtRFC1Δ1-334 | agaGTCGACattatcgagacttccttgcc | cgcGGTACCtctctttctcttggcaccag |
| AtRFC1Δ1-457 | agaGTCGACatggctgccaacttcgatag | cgcGGTACCtctctttctcttggcaccag |
| AtRFC1Δ917-956 | agaGTCGACatgtcggatattaggaagtg | cgcGGTACCttcaaatccaattccacttg |
| AtRFC1Δ897-956 | agaGTCGACatgtcggatattaggaagtg | CgcGGTACCcttttctccatcggttgctt |
| AtRFC2Δ1-224 | agaGTCGACatggtcaaccaagaaaacgt | cgcGGTACCaggtgctttcgctgtttcac |
| AtRFC2Δ314-333 | agaGTCGACatggcgtcttcttcatcaac | cgcGGTACCataagatccaactccatcac |
| AtRFC2Δ294-333 | agaGTCGACatggcgtcttcttcatcaac | cgcGGTACCaagtttcagatactcagcca |
| AtRFC3Δ1-247 | agaGTCGACatgaaggaaattacagagga | cgcGGTACCctttgctgcaccaacaatga |
| AtRFC3Δ350-369 | agaGTCGACatgactgagctaacgtcggc | cgcGGTACCctgcagtttgtcgttgcaac |
| AtRFC3Δ330-369 | agaGTCGACatgactgagctaacgtcggc | cgcGGTACCcagttgaactctgacagcgg |
| AtRFC4Δ1-213 | agaGTCGACatcacatatctgcagagtgc | cgcGGTACCgaagtcttgagccatttctg |
| AtRFC4Δ320-339 | agaGTCGACatggcgccagttcttcagag | cgcGGTACCaagctgcaagtactcatccg |
| AtRFC4Δ300-339 | agaGTCGACatggcgccagttcttcagag | cgcGGTACCgatcttagccttttgcatgt |
| AtRFC5Δ1-239 | cgcTCTAGAatgtatccgttcacaggtaacc | cgcGGATCCcccaaacgttgaaatgagga |
| AtRFC5Δ335-354 | cgcTCTAGAatgttgtgggtcgacaagta | cgcGGATCCtatgtgaaatatggctttct |
