# Supplementary material for: Subunit Interaction Differences Between the Replication Factor C Complexes in Arabidopsis and Rice
Source: Front Plant Sci. 2018 Jun 19;9:779. doi: 10.3389/fpls.2018.00779 (PMC6018503; doi:10.3389/fpls.2018.00779)
Supplement: Supplementary file 5 [file Presentation_1.pdf]

Subunit interaction differences between the replication factor C complexes in *Arabidopsis* and rice

Authors: Yueyue Chen†, Jie Qian†, Li You, Xiufeng Zhang, Jinxia Jiao, Yang Liu, Jie Zhao*

Address: State Key Laboratory of Hybrid Rice, College of Life Sciences, Wuhan University, Wuhan 430072, China

***Corresponding author:** Jie Zhao

† These authors contributed equally to this work.

E-mail: jzhao@whu.edu.cn

Tel: 86-27-68756010

SUPPLEMENTARY MATERIAL

Figure S1. Phylogenetic analysis of eukaryotic RFC subunits. The amino acid sequences of the RFC subunits are downloaded from NCBI (http://www.ncbi.nlm.nih.gov/) and aligned with CLUSTALX. The phylogenetic tree is constructed by the Neighbor-Joining algorithm using the MEGA4 software. At, *Arabidopsis thaliana*; Os, *Oryza sativa*; Sc, *Saccharomyces cerevisiae*; Ce, *Caenorhabditis elegans*; H, *Homo sapiens*; Mm, *Mus musculus*.

Figure S2. Temporal and spatial expressions of *AtRFC2/3/5*. (A-C) qRT-PCR analysis of the AtRFC2/3/5 expressions in various organs of wild-type plants. 1DAP, 1DAP silique; 2DAP, 2DAP silique; 3DAP, 3DAP silique.

Figure S3. Complementation assays of *AtRFC2* and *AtRFC5* mutants. (A) Genotypic analysis of rfc2-1/+ and rfc5-1/+ mutants, and genotypic confirmation of homozygous transgenic mutant plants. FP, forward primer; RP, reverse primer; LBb1.3 and CSLB, primer at left border of the vector. (B) Phenotypes of the siliques in the homozygosis transformed complemented (com) plants based on the rfc2-1/+

and rfc5-1/+ mutants. Ar, the seed abortion rate; n, the total number of counted seeds.

Figure S4. Subcellular localization of OsRFC2 and OsRFC3 in the stably transformed rice callus cells. (A) Fluorescent signal of *35S::GFP* is distributed in both cytoplasm and nucleus. (B, C) *35S::OsRFC2/3-GFP* are mainly localized in the nucleus, and a small amount in the cytoplasm of the callus cells. Bars=30 μm.

Figure S5. The conservative substitution of AtRFC1 and OsRFC1. Confocal images of tobacco leaf cells expressing transiently the indicated fusion proteins. (A-J) AtRFC1 can interact with OsRFC2/3/4/5 in the presence of all other three subunits. (K-T) OsRFC1 can directly interact with AtRFC2/3/4/5. Bars=30 μm.

Figure S6. The conservative substitution of AtRFC2/3/4/5 and OsRFC2/3/4/5. Confocal images of tobacco leaf cells expressing transiently the indicated fusion proteins. (A-C) OsRFC2-YN can interact with AtRFC2/3/5-YC. (D, E) OsRFC3-YN can interact with AtRFC2/4-YC. (F, G) OsRFC4-YN can interact with AtRFC3/4-YC. (H) OsRFC5-YN can interact with AtRFC4-YC. YC, YFP C-terminal fragment (aa 156-239); YN, YFP N-terminal fragment (aa 1-155). Bars=30 μm.

Figure S7. Interaction assay between the truncated AtRFC1 protein and AtRFC2/3/4/5. (A-D) AtRFC1 Δ1-334 interacts with AtRFC2/3/4/5, respectively. (E-H) AtRFC1 Δ1-457 interacts with AtRFC2/3/4/5, respectively. (I-L) AtRFC1 Δ917-956 interacts with AtRFC2/3/4/5, respectively. (M-P) AtRFC1 Δ897-956 interacts with AtRFC2/3/4/5, respectively. Bars=30 μm.

Figure S8. Interaction assay between the truncated AtRFC2 protein and AtRFC1/3/4. Confocal images of tobacco leaf cells expressing transiently the indicated fusion proteins. (A) Interaction between AtRFC2 Δ1-224 and AtRFC1 in the presence of AtRFC3/4/5. (B, C) Interactions between AtRFC2 Δ1-224 and AtRFC3/4. (D) Interaction between AtRFC2 Δ314-333 and AtRFC1 in the presence of AtRFC3/4/5. (E, F) Interactions between AtRFC2 Δ314-333 and AtRFC3/4. (G) Interaction between AtRFC2 Δ294-333 and AtRFC1 in the presence of AtRFC3/4/5. (H, I) Interactions between AtRFC2 Δ294-333 and AtRFC3/4. YFP^C, YFP C-terminal

fragment (aa 156-239); YFP^N, YFP N-terminal fragment (aa 1-155). Bars=30 μm.

Figure S9. Interaction assay between the truncated AtRFC3 protein and AtRFC1/2/4. Confocal images of tobacco leaf cells expressing transiently the indicated fusion proteins. (A) Interaction between AtRFC3 Δ1-247 and AtRFC1 in the presence of AtRFC2/4/5. (B, C) Interactions between AtRFC3 Δ1-247 and AtRFC2/4. (D) Interaction between AtRFC3 Δ350-369 and AtRFC1 in the presence of AtRFC2/4/5. (E, F) Interactions between AtRFC3 Δ350-369 and AtRFC2/4. (G) Interaction between AtRFC3 Δ330-369 and AtRFC1 in the presence of AtRFC2/4/5. (H, I) Interactions between AtRFC3 Δ330-369 and AtRFC2/4. YFP^C, YFP C-terminal fragment (aa 156-239); YFP^N, YFP N-terminal fragment (aa 1-155). Bars=30 μm.

Figure S10. Interaction assay between the truncated AtRFC4 protein and AtRFC1/2/3/5. Confocal images of tobacco leaf cells expressing transiently the indicated fusion proteins. (A) Interaction between AtRFC4 Δ1-213 and AtRFC1 in the presence of AtRFC2/3/5. (B-D) Interactions between AtRFC4 Δ1-213 and AtRFC2/3/5. (E) Interaction between AtRFC4 Δ320-339 and AtRFC1 in the presence of AtRFC2/3/5. (F-H) Interactions between AtRFC4 Δ320-339 and AtRFC2/3/5. (I) Interaction between AtRFC4 Δ300-339 and AtRFC1 in the presence of AtRFC2/3/5. (J-L) Interactions between AtRFC4 Δ300-339 and AtRFC2/3/5. YFP^C, YFP C-terminal fragment (aa 156-239); YFP^N, YFP N-terminal fragment (aa 1-155). Bars=30 μm.

Figure S11. Interaction assay between the truncated AtRFC5 protein and AtRFC1/4. Confocal images of tobacco leaf cells expressing transiently the indicated fusion proteins. (A) Interaction between AtRFC5 Δ1-239 and AtRFC1 in the presence of AtRFC2/3/4. (B) Interaction between AtRFC5 Δ1-239 and AtRFC4. (C) Interaction between AtRFC5 Δ335-354 and AtRFC1 in the presence of AtRFC2/3/4. (D) Interaction between AtRFC5 Δ335-354 and AtRFC4. YFP^C, YFP C-terminal fragment (aa 156-239); YFP^N, YFP N-terminal fragment (aa 1-155). Bars=30 μm.

Figure S12. Interaction assay between the truncated OsRFC1 protein and OsRFC2/3/4/5. Confocal images of tobacco leaf cells expressing transiently the indicated fusion proteins. (A-D) Interactions between OsRFC1 Δ1-642 and

OsRFC2/3/4/5. (E-H) Interactions between OsRFC1 Δ1-721 and OsRFC2/3/4/5. (I-L) Interactions between OsRFC1 Δ722-1021 and OsRFC2/3/4/5. (M-P) Interactions between OsRFC1 Δ640-1021 and OsRFC2/3/4/5. YFP^C, YFP C-terminal fragment (aa 156-239); YFP^N, YFP N-terminal fragment (aa 1-155). Bars=30 μm.

Figure S13. Interaction assay between the truncated OsRFC2 protein and OsRFC1/3/5. Confocal images of tobacco leaf cells expressing transiently the indicated fusion proteins. (A-C) Interactions between OsRFC2 Δ1-221 and OsRFC1/3/5. (D-F) Interactions between OsRFC2 Δ320-339 and OsRFC1/3/5. (G-I) Interactions between OsRFC2 Δ300-339 and OsRFC1/3/5. (J-L) Interactions between OsRFC2 Δ240-339 and OsRFC1/3/5. YFP^C, YFP C-terminal fragment (aa 156-239); YFP^N, YFP N-terminal fragment (aa 1-155). Bars=30 μm.

Figure S14. Interaction assay between the truncated OsRFC3 protein and OsRFC1/2/4. Confocal images of tobacco leaf cells expressing transiently the indicated fusion proteins. (A-C) Interactions between OsRFC3 Δ1-245 and OsRFC1/2/4. (D-F) Interactions between OsRFC3 Δ342-361 and OsRFC1/2/4. (G-I) Interactions between OsRFC3 Δ322-361 and OsRFC1/2/4. (J-L) Interactions between OsRFC3 Δ62-361 and OsRFC1/2/4. YFP^C, YFP C-terminal fragment (aa 156-239); YFP^N, YFP N-terminal fragment (aa 1-155). Bars=30 μm.

Figure S15. Interaction assay between the truncated OsRFC4 protein and OsRFC1/3, between the truncated OsRFC5 protein and OsRFC1/2. Confocal images of tobacco leaf cells expressing transiently the indicated fusion proteins. (A, B) Interactions between OsRFC4 Δ1-222 and OsRFC1/3. (C, D) Interactions between OsRFC4 Δ216-335 and OsRFC1/3. (E, F) Interactions between OsRFC4 Δ136-335 and OsRFC1/3. (G, H) Interactions between OsRFC4 Δ36-335 and OsRFC1/3. (I, J) Interactions between OsRFC5 Δ1-237 and OsRFC1/2. (K, L) Interactions between OsRFC5 Δ1-300 and OsRFC1/2. (M, N) Interactions between OsRFC5 Δ235-354 and OsRFC1/2. (O, P) Interactions between OsRFC5 Δ205-354 and OsRFC1/2. (Q, R) Interactions between OsRFC5 Δ155-354 and OsRFC1/2.YFP^C, YFP C-terminal fragment (aa 156-239); YFP^N, YFP N-terminal fragment (aa 1-155). Bars=30 μm.
