# Supplementary figures and images for: Subunit Interaction Differences Between the Replication Factor C Complexes in Arabidopsis and Rice
Source: Front Plant Sci. 2018 Jun 19;9:779. doi: 10.3389/fpls.2018.00779 (PMC6018503; doi:10.3389/fpls.2018.00779)

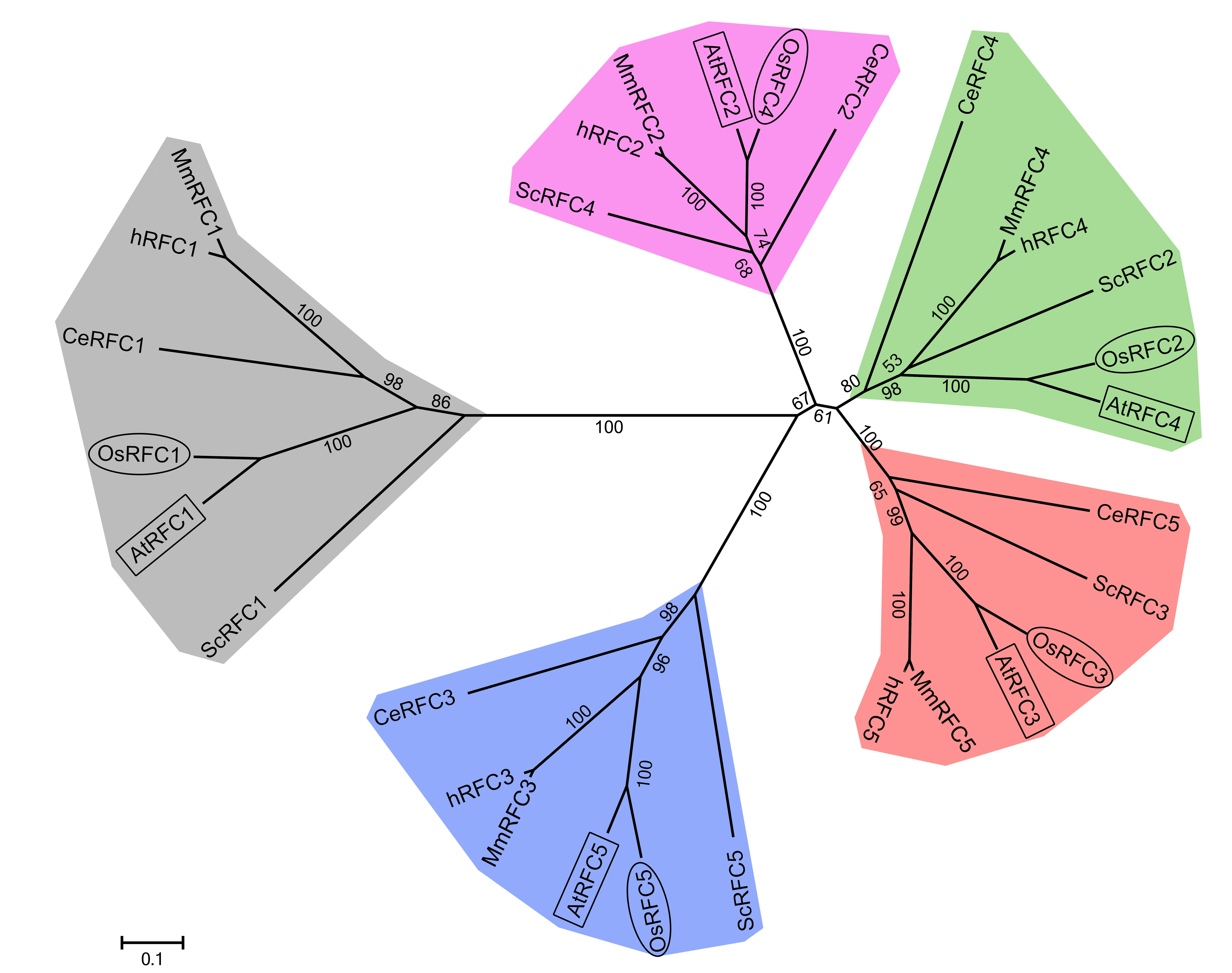

Supplement: Supplementary file 6 [file Image_1.JPEG]

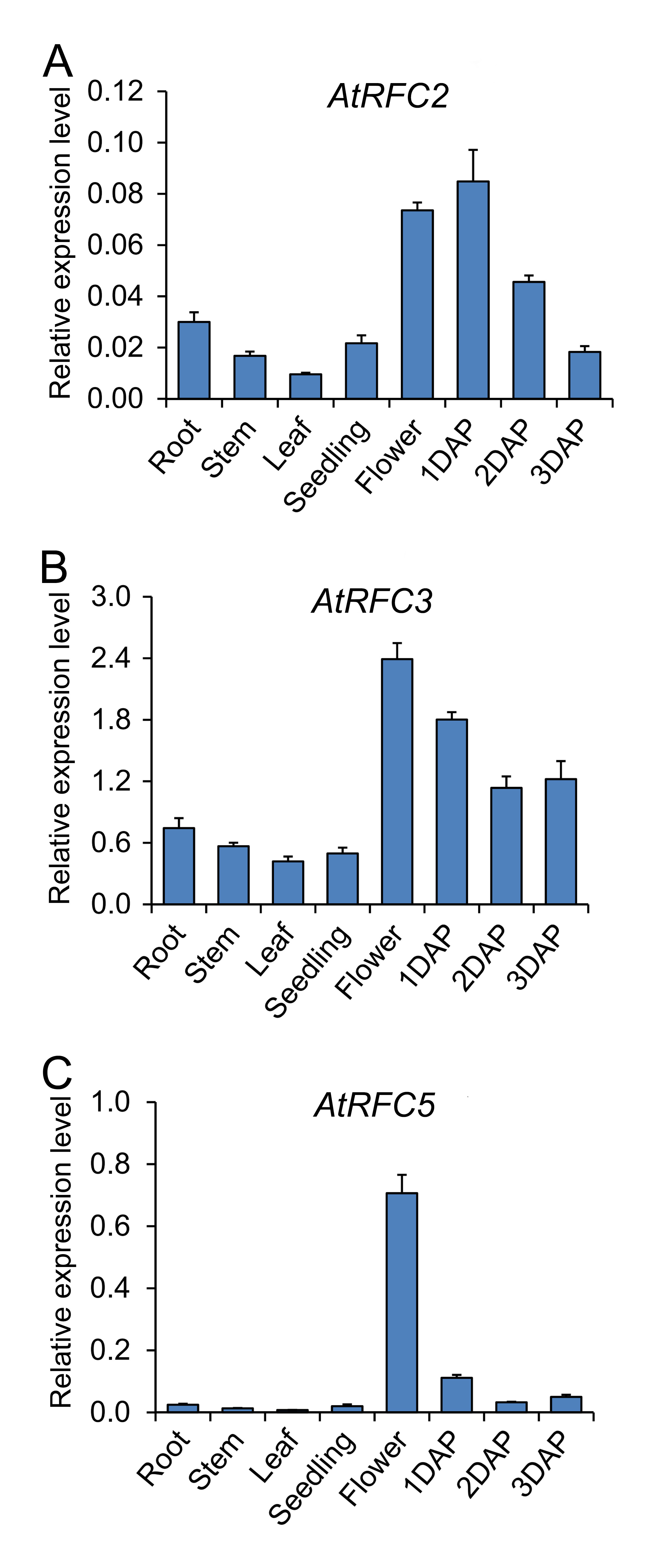

Supplement: Supplementary file 7 [file Image_2.JPEG]

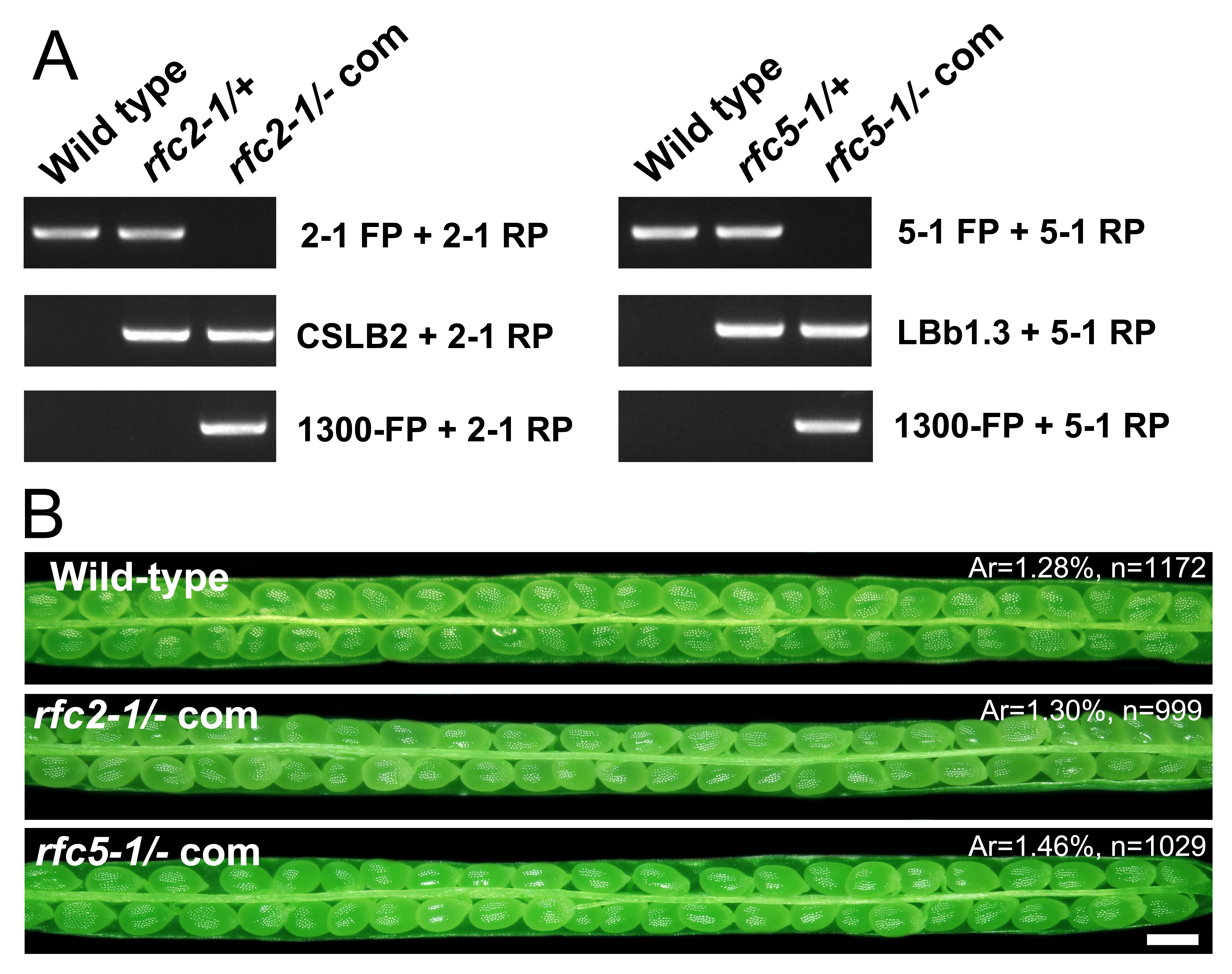

Supplement: Supplementary file 8 [file Image_3.JPEG]

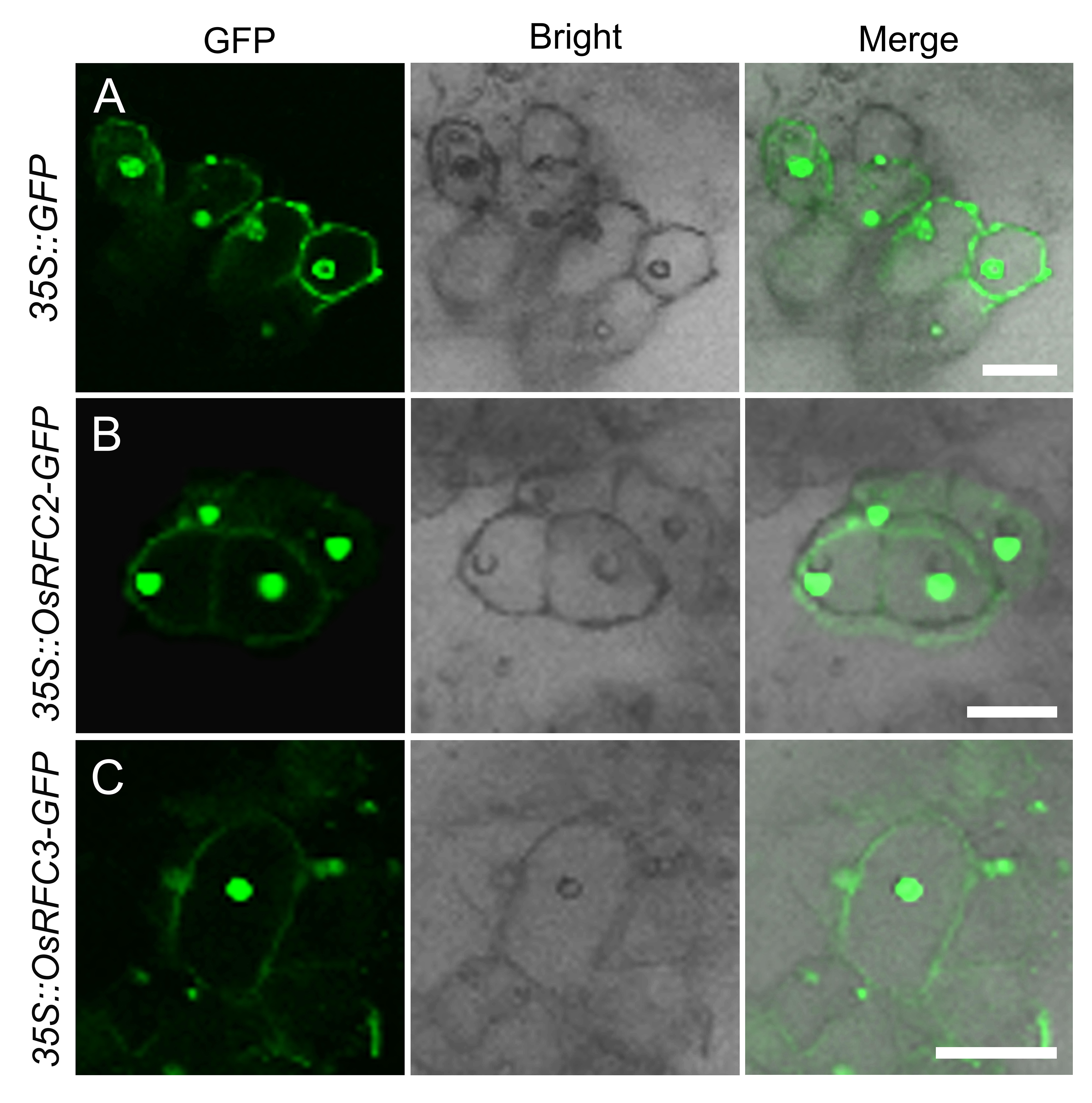

Supplement: Supplementary file 9 [file Image_4.JPEG]

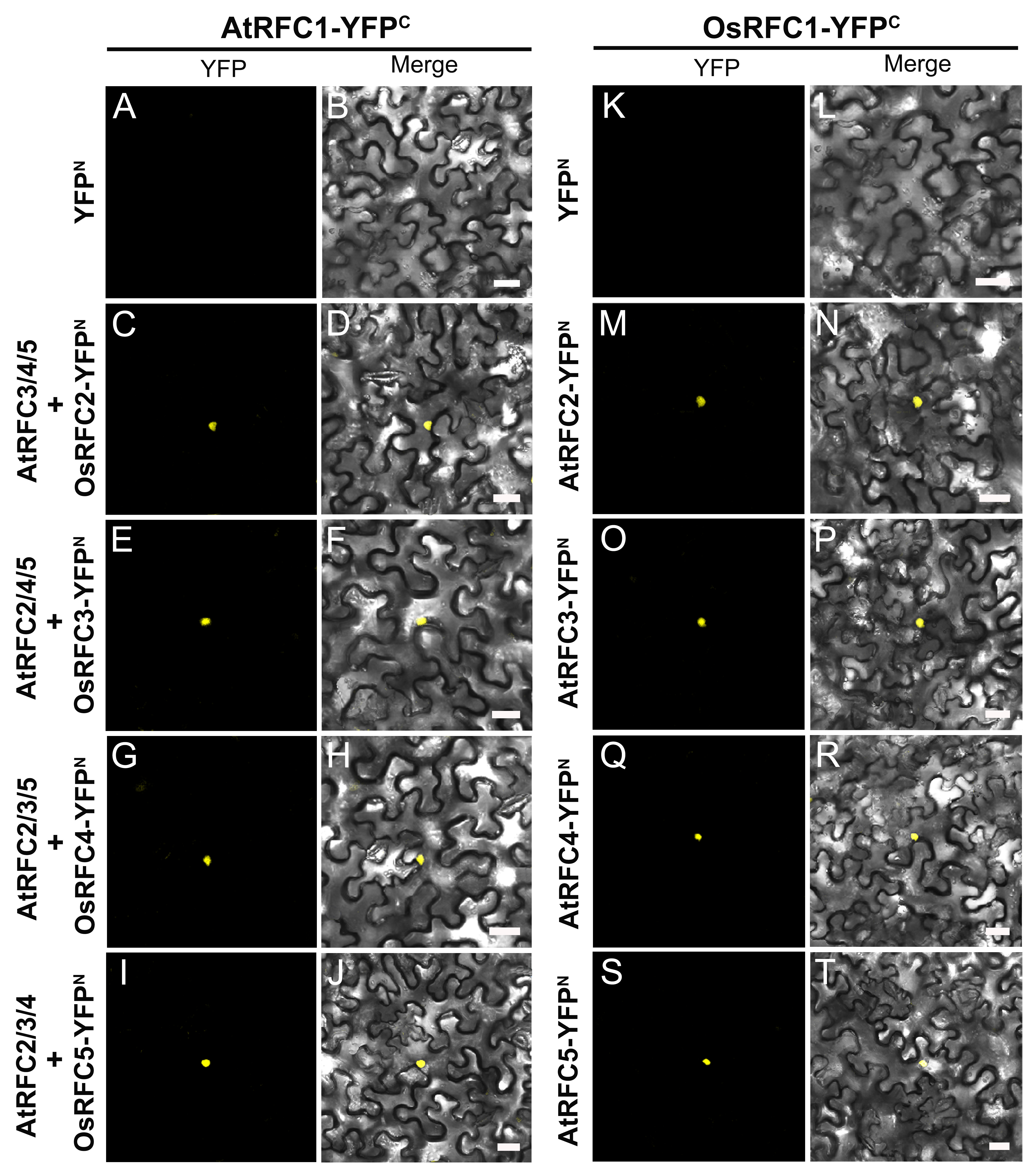

Supplement: Supplementary file 10 [file Image_5.JPEG]

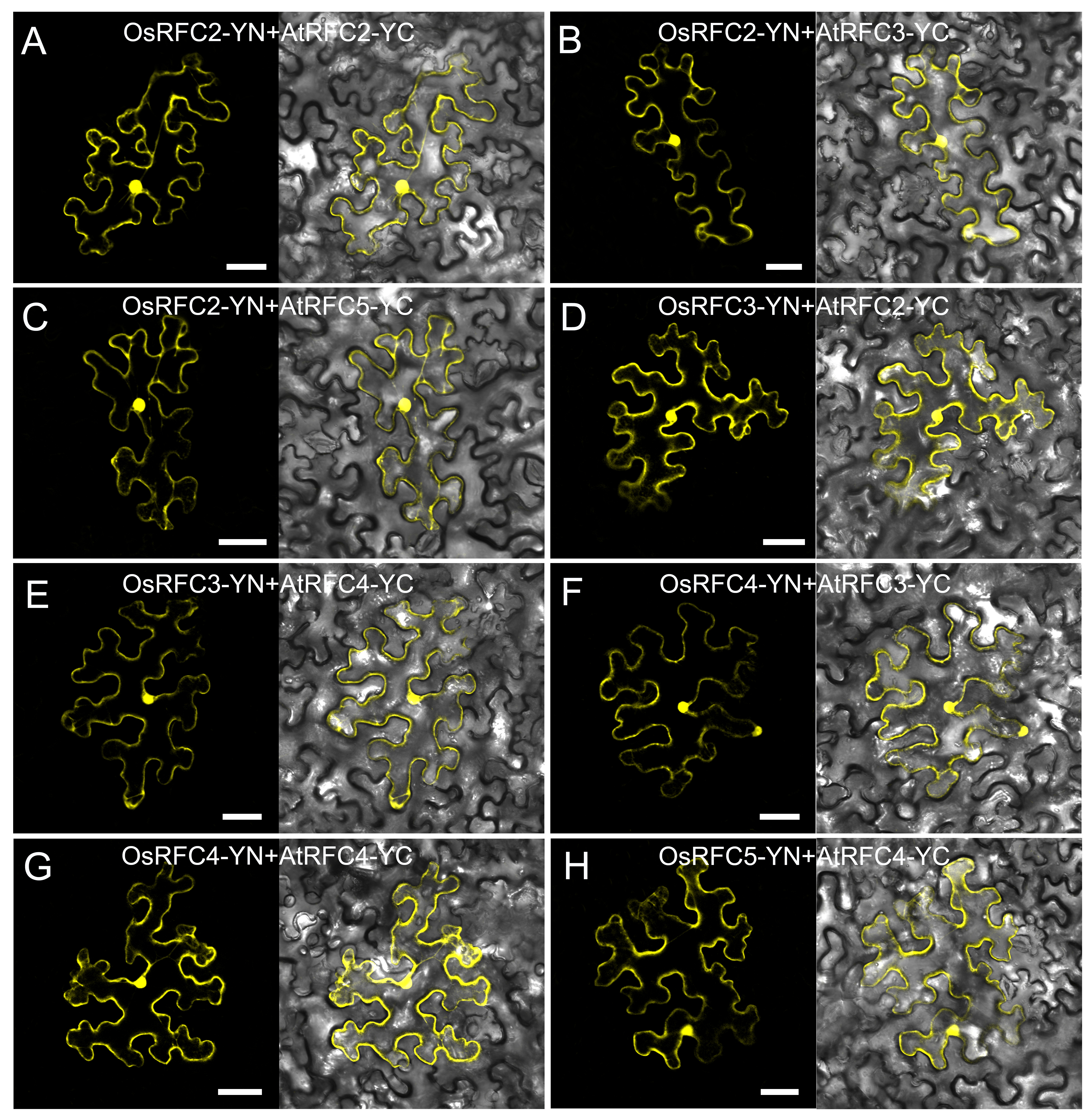

Supplement: Supplementary file 11 [file Image_6.JPEG]

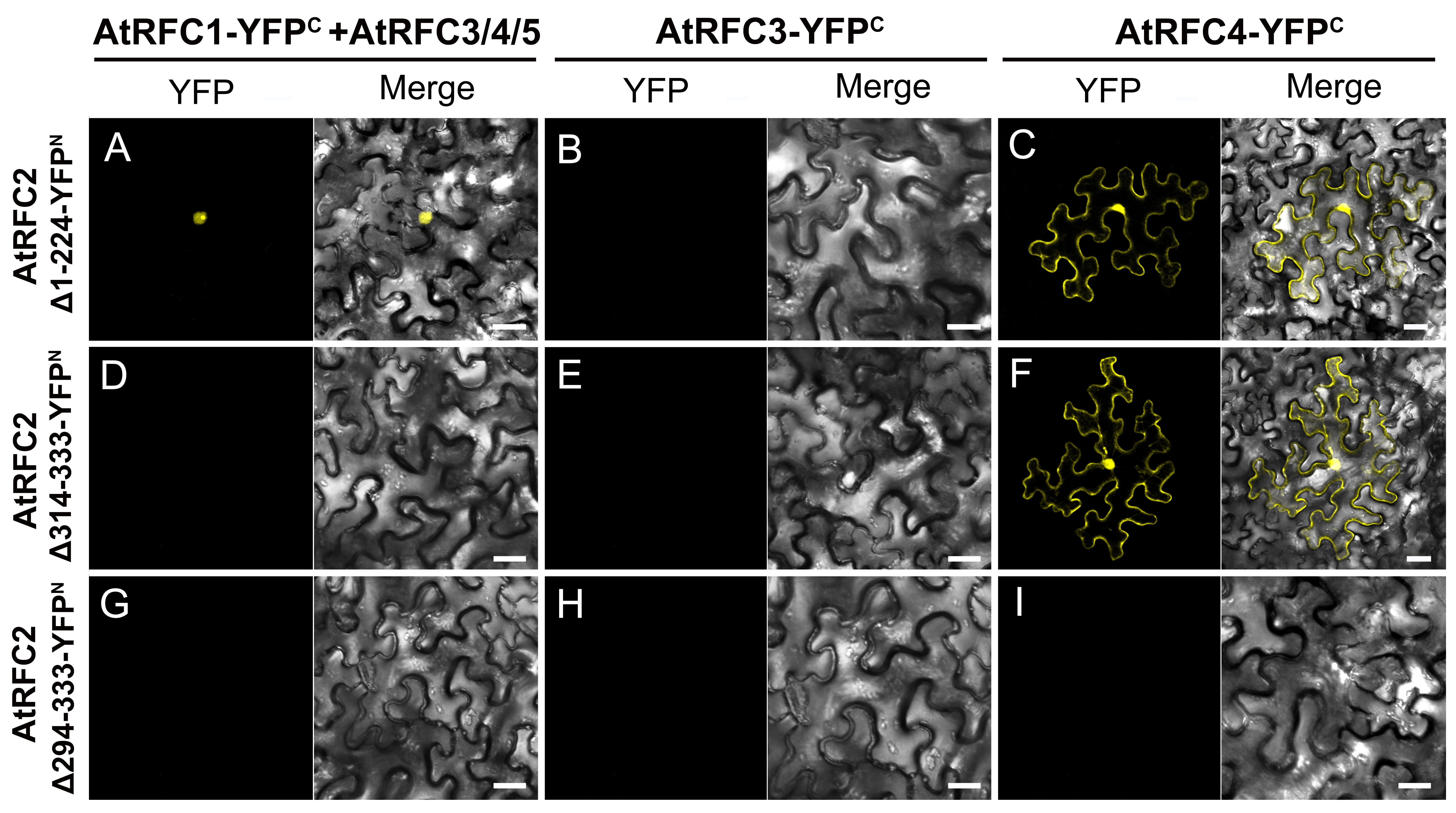

Supplement: Supplementary file 13 [file Image_8.JPEG]

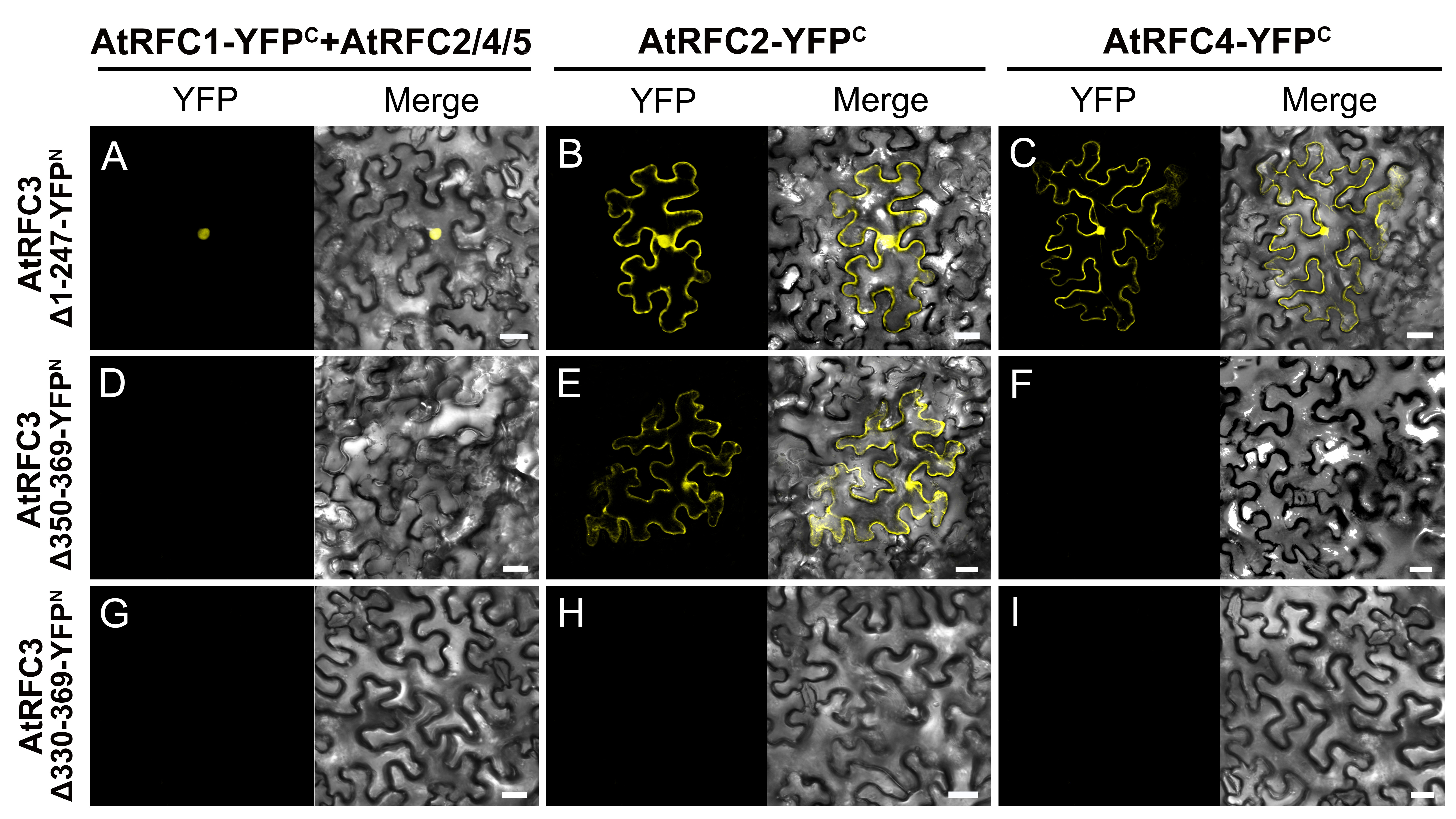

Supplement: Supplementary file 14 [file Image_9.JPEG]

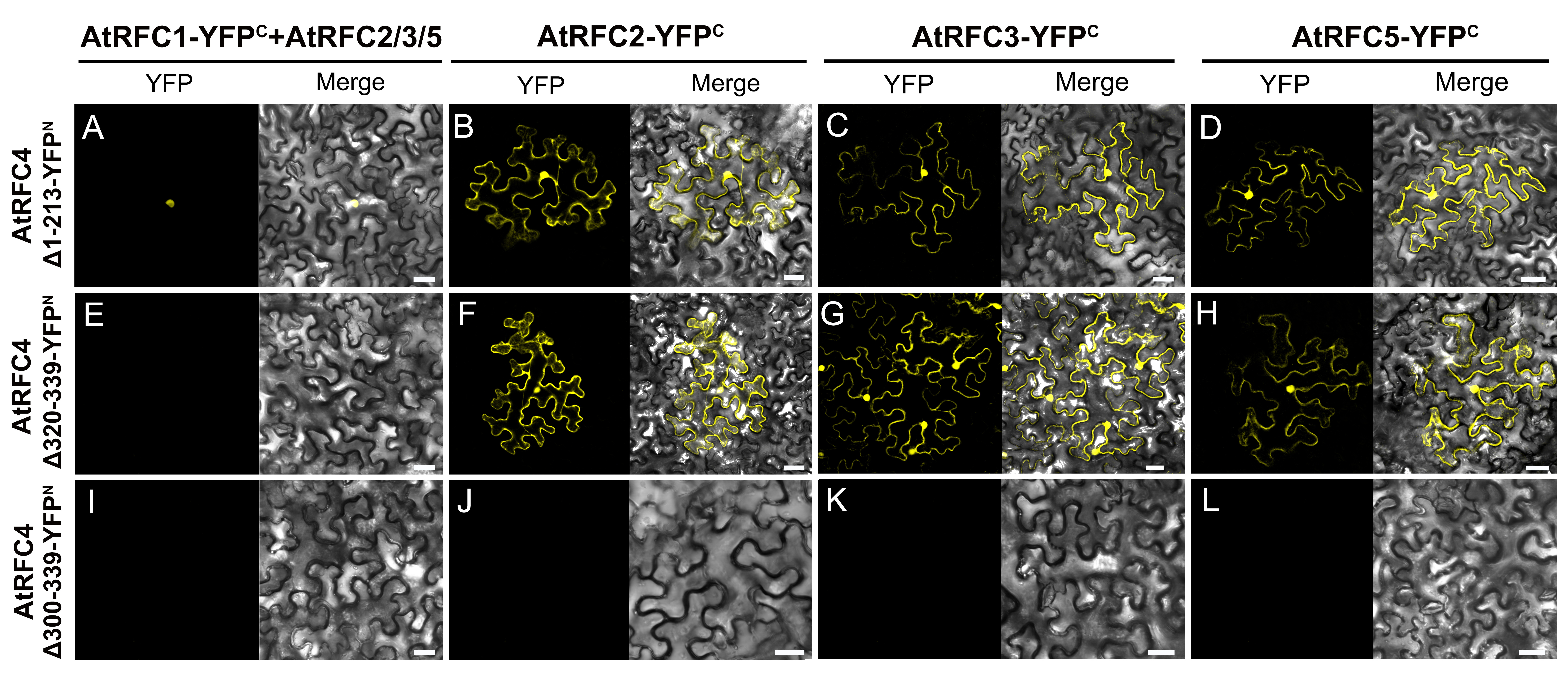

Supplement: Supplementary file 15 [file Image_10.JPEG]

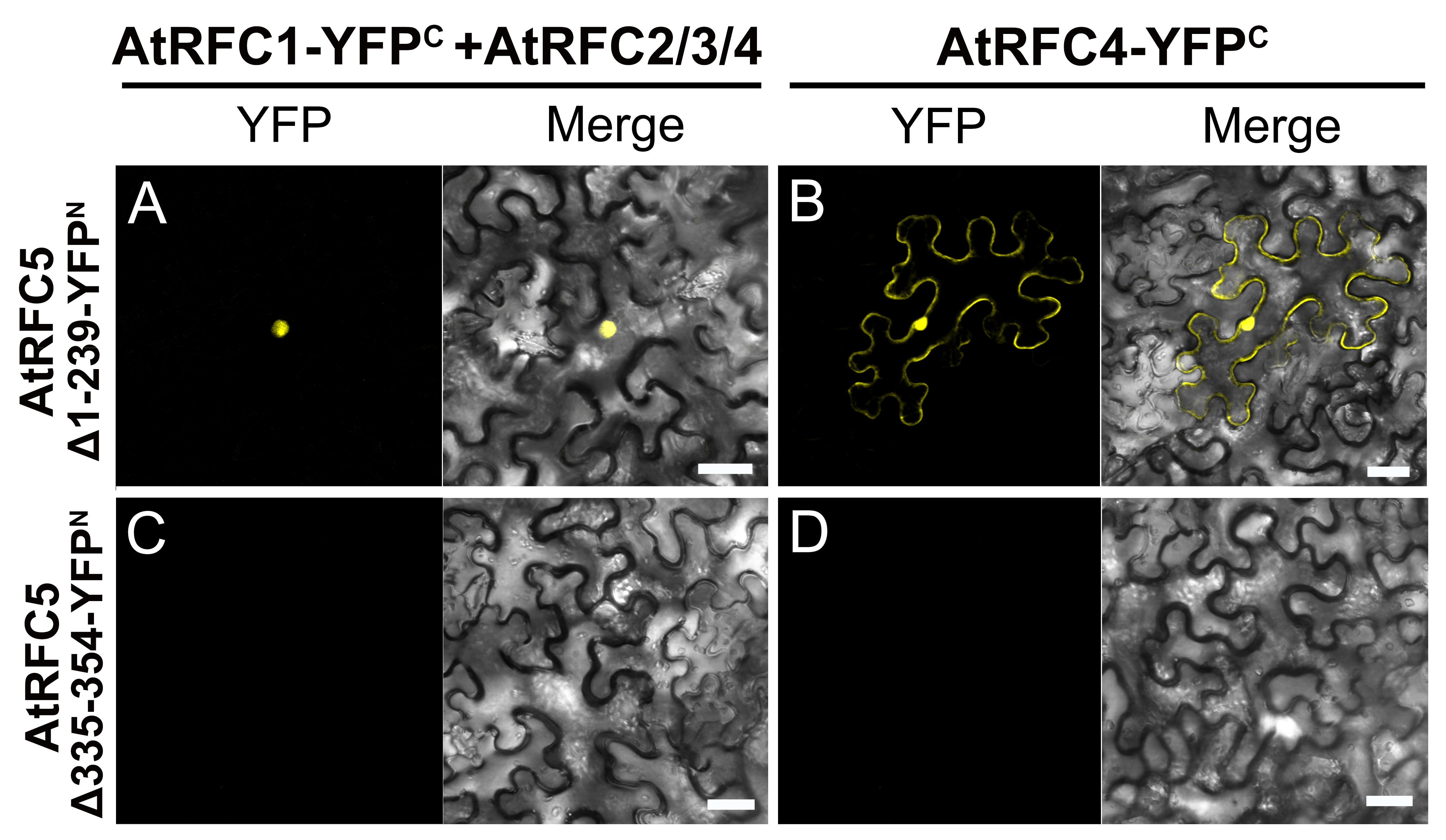

Supplement: Supplementary file 16 [file Image_11.JPEG]

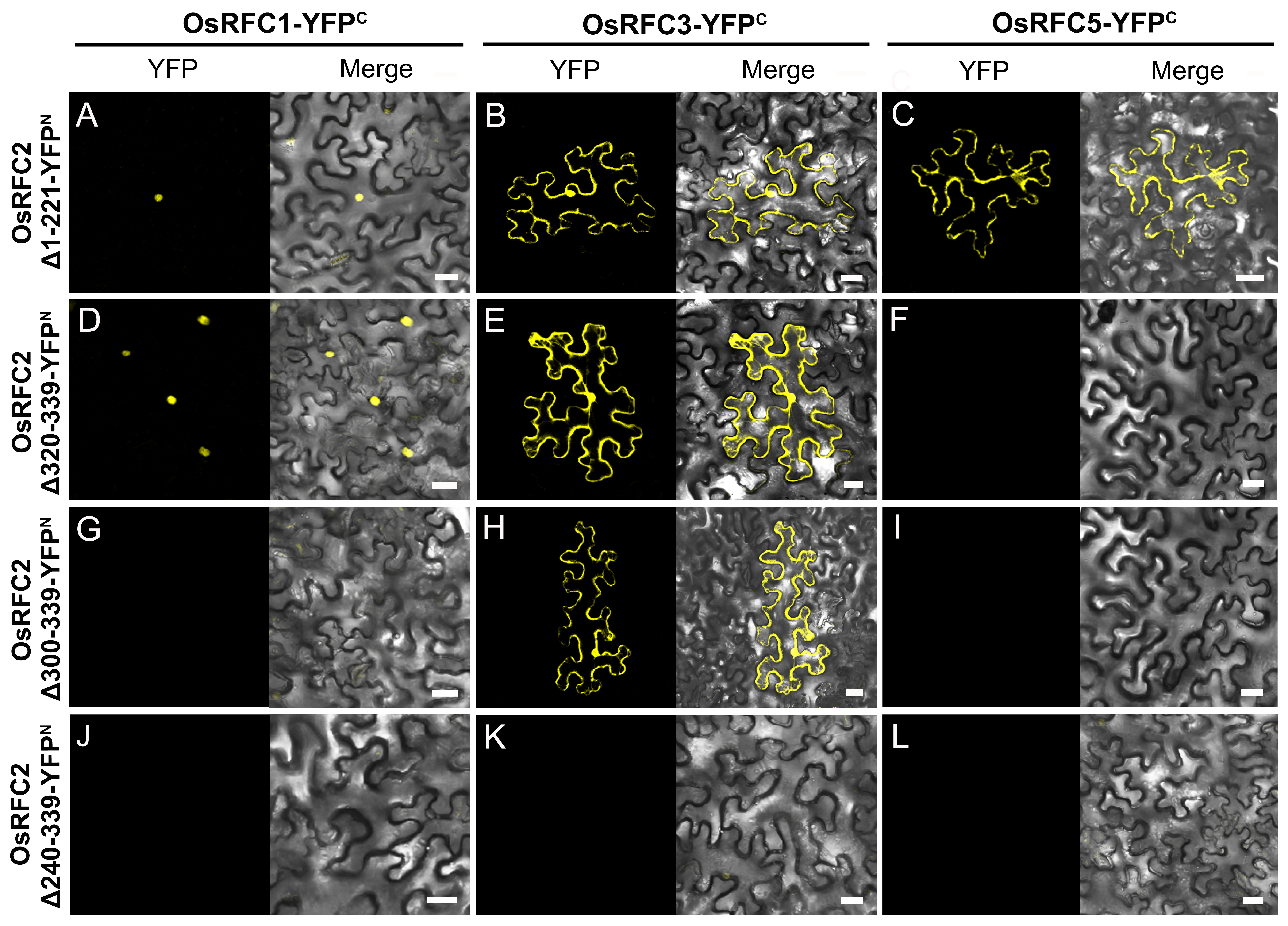

Supplement: Supplementary file 18 [file Image_13.JPEG]

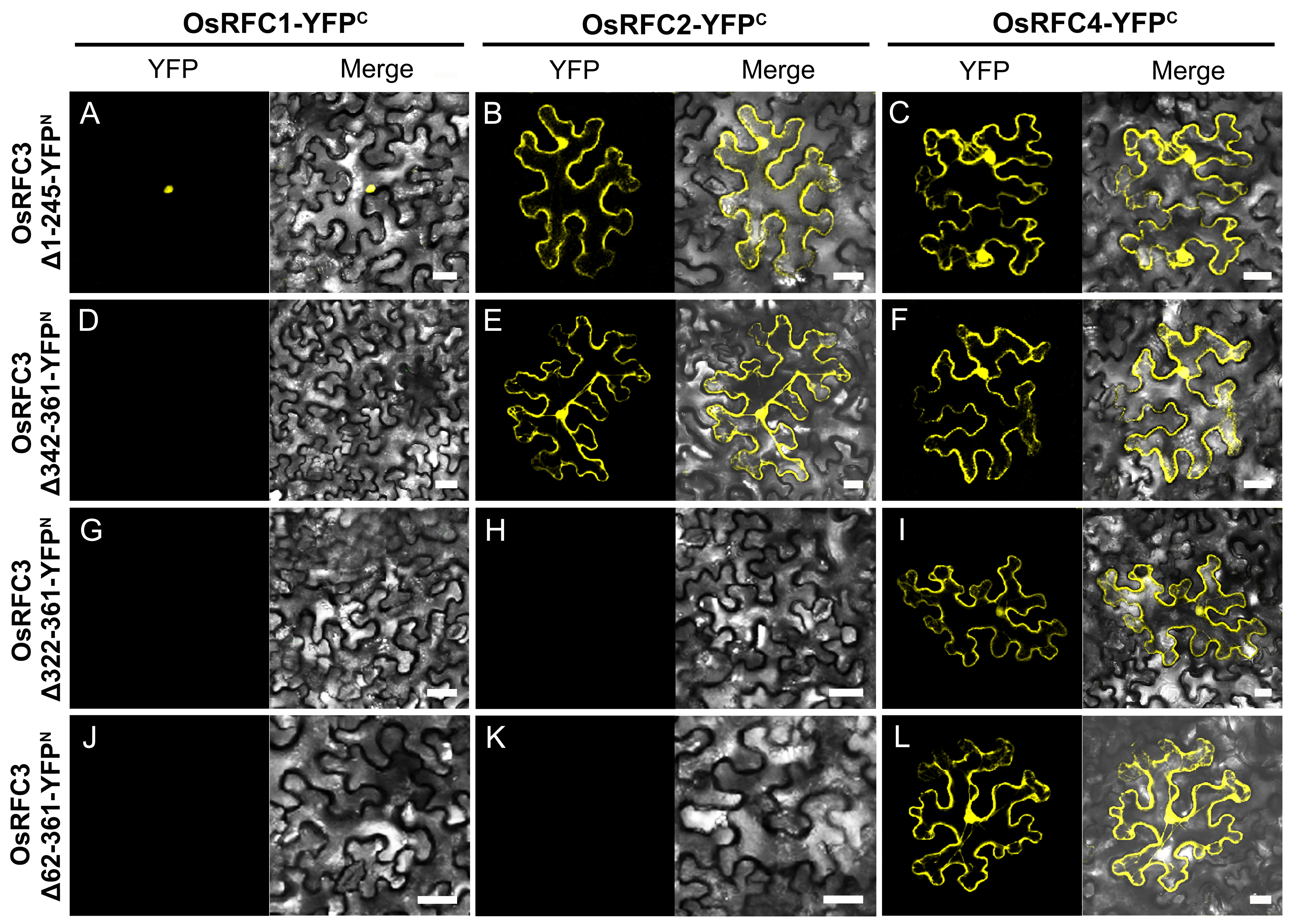

Supplement: Supplementary file 19 [file Image_14.JPEG]

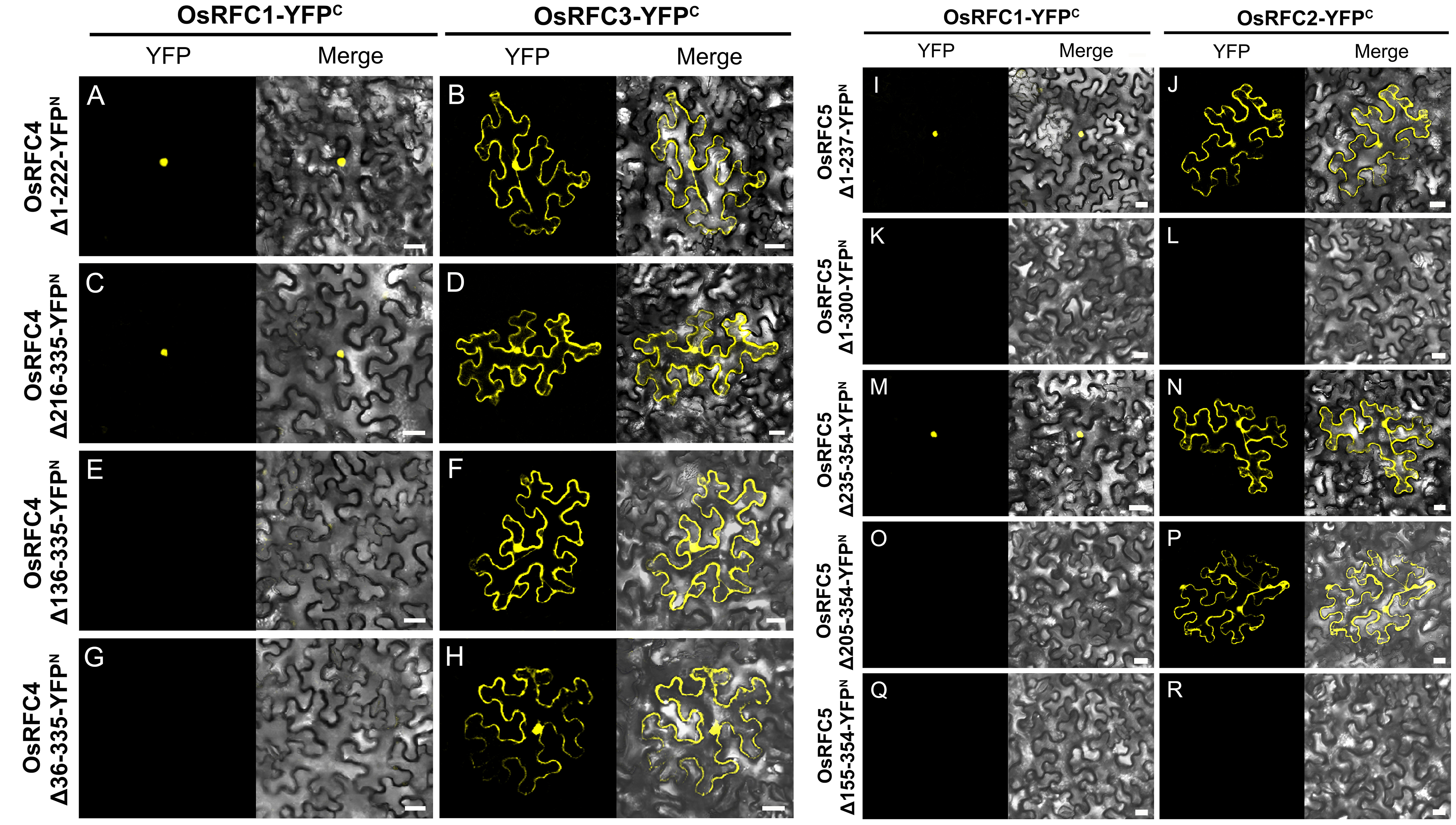

Supplement: Supplementary file 20 [file Image_15.JPEG]
